# Identification and characterization of HAK/KUP/KT potassium transporter gene family in barley and their expression under abiotic stress

**DOI:** 10.1186/s12864-021-07633-y

**Published:** 2021-05-01

**Authors:** Kangfeng Cai, Fanrong Zeng, Junmei Wang, Guoping Zhang

**Affiliations:** 1 Institute of Crop and Nuclear Technology Utilization, Zhejiang Academy of Agricultural Sciences, Hangzhou, 310021 China; 2 Institute of Crop Science, Zhejiang University, Hangzhou, 310058 China

**Keywords:** HAK/KUP/KT, Gene family, Barley, Gene expression, Abiotic stress response

## Abstract

**Background:**

HAK/KUP/KT (High-affinity K^+^ transporters/K^+^ uptake permeases/K^+^ transporters) is the largest potassium transporter family in plants, and plays pivotal roles in K^+^ uptake and transport, as well as biotic and abiotic stress responses. However, our understanding of the gene family in barley (*Hordeum vulgare* L.) is quite limited.

**Results:**

In the present study, we identified 27 barley *HAK/KUP/KT* genes (hereafter called *HvHAKs*) through a genome-wide analysis. These *HvHAKs* were unevenly distributed on seven chromosomes, and could be phylogenetically classified into four clusters. All HvHAK protein sequences possessed the conserved motifs and domains. However, the substantial difference existed among *HAK* members in *cis*-acting elements and tissue expression patterns. Wheat had the most orthologous genes to barley *HAKs*, followed by *Brachypodium distachyon*, rice and maize. In addition, six barley *HAK* genes were selected to investigate their expression profiling in response to three abiotic stresses by qRT-PCR, and their expression levels were all up-regulated under salt, hyperosmotic and potassium deficiency treatments.

**Conclusion:**

Twenty seven *HAK* genes (*HvHAKs*) were identified in barley, and they differ in tissue expression patterns and responses to salt stress, drought stress and potassium deficiency.

**Supplementary Information:**

The online version contains supplementary material available at 10.1186/s12864-021-07633-y.

## Background

Potassium (K) is the second most abundant mineral nutrient in plants, comprising 2–10% of plant dry weight [1]. K is not only essential for plants to maintain normal physiological and biochemical processes such as stomatal movement, photosynthesis, osmoregulation, protein synthesis, enzyme activation, but also involved in the responses to biotic and abiotic stresses [2, 3]. K in soil exists in several different states/pools [4, 5], and available K for plants is commonly quite low due to the strong K adsorption by 2:1 silicate minerals [4]. In plant cells, the cytosolic K^+^ concentration is relatively constant, ranging from 60 to 200 mM, while vacuolar K^+^ concentration is more variable, ranging from 20 mM (in K^+^-deficient plants) to 500 mM (in specialized guard cells) [1, 6, 7]. In addition, apoplastic K^+^ concentration also shows a large variation, ranging from 10 to 200 mM or even up to 500 mM [8]. In short, K concentration in plants differs greatly between species, tissues, and cell organs.

As sessile organisms, plants have evolved the efficient K^+^ transport systems to maintain optimal growth under the conditions of highly variable K^+^ levels [9]. In plants, K^+^ is first taken up by roots, and then translocated to aerial parts and distributed within cells into different compartments [10]. All these processes are involved in K^+^ transmembrane transport, which is achieved mainly by K^+^ channels and transporters. So far, K^+^ uptake systems are better identified and characterized in *Arabidopsis thaliana* than in any other plant species. Currently it is well documented that non-selective cation channels (NSCC) probably play the major role in K^+^ uptake at high concentrations (> 10 mM), while the low-affinity inward rectifying channel AtAKT1 dominates K^+^ uptake at intermediate concentrations (1 mM). Under lower external K^+^ concentrations (100 μM), high-affinity potassium transporter AtHAK5, and together with AtAKT1, contributes to K^+^ acquisition. AtHAK5 is the only transporter capable of taking up K^+^ under extremely low concentrations (< 10 μM) [9]. Thus, it is imperative to characterize the function and expression pattern of high-affinity potassium transporters.

There are three families of K^+^ transporters in plants: HAK/KUP/KT (High-affinity K^+^ transporters/K^+^ uptake permeases/K^+^ transporters), HKT (High-affinity K^+^ transporters) and CPA (monovalent cation/proton antiporters) [10]. HKT transporters function as K^+^/Na^+^ co-transporters or Na^+^ transporters [11]. CPA family can be further divided into NHX (Na^+^/H^+^ exchangers), CHX (Cation/H^+^ exchangers) and KEA (K^+^ efflux antiporters) subfamilies [10], and only several members (viz. AtCHX17, AtKEA4, AtKEA5 and AtKEA6) have been proved to function in K^+^ transport [12, 13]. The HAK/KUP/KT members in bacteria and fungi were named as KUP (K^+^ uptake permease) and HAK (High-affinity K^+^ transporter), respectively [14, 15]. Diverse acronyms were assigned when the family members in plants were identified, viz. HAK for barley [16], KT (K^+^ transporter) or KUP for *Arabidopsis* [17–19], so composite names HAK/KUP/KT or KT/HAK/KUP or KT/KUP/HAK were widely used for the whole transporter family in plants [10, 20, 21]. HAK/KUP/KT (HAK hereafter) is the largest K^+^ transporter family, and its members are predicted as K^+^/H^+^ symporters [22]. *HAK* genes are absent in animal cells, but present in all known plant genomes [21], and first identified in barley [16] and *Arabidopsis* [17–19], and later also in rice [23, 24], wheat [25] and maize [26]. However, the mechanisms of HAK transporters in mediating K^+^ uptake and transport are still completely unclear.

HAK transporters play the diverse roles in K^+^ uptake and translocation, salt and drought stress response, as well as morphological development of root and shoot [21]. In *Arabidopsis*, AtHAK5 is a major K^+^ deprivation-induced high-affinity K^+^ uptake transporter, and is the only system being able to take up K^+^ at external concentrations below 10 μM [9, 27]. AtKUP7 is responsible for K^+^ uptake at high K^+^ concentrations, and may be also involved in K^+^ transport into xylem sap, affecting K^+^ translocation from roots to shoots [28]. In rice, the expression of *OsHAK1* is up-regulated by K^+^ deficiency in roots. Knockout of *OsHAK1* reduces total K^+^ uptake by approximately 80 and 65% at 0.05–0.1 mM and 1 mM of K^+^ concentrations, respectively, while overexpression of *OsHAK1* increases K^+^ uptake and K^+^/Na^+^ ratio [29]. *OsHAK5* is expressed highly in root epidermis, stele and vascular tissues, and plays major roles not only in K^+^ acquisition, but also in K^+^ upward translocation from roots to shoots under low external K^+^ conditions [22]. K^+^ homeostasis and K^+^/Na^+^ balance are severely disturbed when plants are exposed to salt stress, as high Na^+^ concentrations suppress the expression of *HAK* genes at low K^+^ concentrations [30, 31]. However, AtHAK5 also plays a major role in maintaining high-affinity K^+^ acquisition and plant growth under salt stress [31]. *OsHAK1* is up-regulated by salt stress under normal K^+^ supply condition, but down-regulated at low K^+^ concentrations [29]. The net K^+^ uptake rate of *oshak1* mutant was almost completely blocked by salt stress when K^+^ concentration was below 0.05 mM, demonstrating that *OsHAK1* plays pivotal role in enhancing salt tolerance in rice [29]. Overexpression of *OsHAK5* also increases shoot K^+^/Na^+^ ratio and salt tolerance in rice [22]. Moreover, *OsHAK1* knockout mutants reduce drought tolerance and growth at both vegetative and reproductive stages, while the over-expressed lines enhance drought tolerance and increase grain yield by 35% relative to wild type under drought condition [32]. In addition to K^+^ uptake and translocation, and stress tolerance, some *HAK* genes are also associated with tissue (root and shoot) morphology. It has been reported that *AtHAK5* is the downstream target of ARF2 (Auxin response factor 2) and its overexpression results in longer primary roots under low K^+^ conditions [33]. AtKUP4/TRH1 (Tiny root hair 1) is required for polar localization of PIN1 (An auxin efflux transporter) in root apex, and for gravitropic responses, as well as normal initiation and formation of root hairs [34–37]. AtKUP2/SHY3 (Short hypocotyl 3) mutation affects cell expansion and leads to developmental defects in shoots [38]. In short, the versatile roles of *HAK* genes have been recognized, including K^+^ transport and abiotic stress response, but little has been known about the relevant molecular mechanisms.

Barley (*Hordeum vulgare*) ranks the fourth largest cereal crop worldwide with multiple use as animal feed, human food and brewing material [39]. In comparison with other cereal crops including rice, wheat and maize, barley is well known for its higher salt tolerance, which is largely attributed to its more K^+^ uptake in roots, more Na^+^ exclusion and vacuolar sequestration [40–42]. Although genome-wide identification of *HAK* gene family has been accomplished in rice [24, 43], wheat [25], maize [26], cassava [44], peach [45], pear [46], and willow [47], the similar research has not been done in barley. The release of barley genome data [48] and recently improved annotated reference genome assembly [49] allow it possible to perform genome-wide identification of *HAK* gene family in barley. In the present study, we identified 27 *HAK* genes (*HvHAKs*) from barley genome, and analyzed their phylogenetic relationships, conserved motifs and domains, gene structure, *cis*-acting elements, syntenic relationships, tissue expression patterns and expression profiling in responses to salt stress, osmotic stress and potassium deficiency.

## Results

### Identification of *HvHAKs* in barley

A total of 27 *HvHAK* genes were identified (Table 1; Additional file 1) using barley genome data [48] and recently improved annotated reference genome assembly [49]. The 27 *HvHAK* genes were unevenly distributed on the seven chromosomes, with chromosome 2 containing eight genes, chromosomes 3 and 7 each containing four genes, chromosomes 1, 5 and 6 each containing three genes, and chromosome 4 containing two genes (Additional file 2). *HAK* genes in barley were named in the order of their locations on chromosomes (viz. *HvHAK1* to *HvHAK27*), and no tandem duplication event was observed (Table 1; Additional file 2). The length of HvHAK transporters varied from 724 to 875 amino acids (aa), and the number of transmembrane segments ranged from 10 to 14, with 11–12 (66.7%) being the most abundant (Table 1). The isoelectric points (pI) of HvHAK proteins ranged from 5.65 to 8.99, with only 6 members being below 7 (Table 1). The theoretical molecular weights (MW) of HvHAK proteins were in the range of 79.93 to 98.04 kDa. All the HvHAK transporters contained a typical “k_trans” domain (PF02705), which is specific to HAK potassium transporter family members (Table 1). In addition, all HvHAKs were localized on plasma membrane (Table 1).

**Table 1:** Twenty seven *HvHAK* genes identified in barley

Name	Gene ID	Length (aa)	Intron	TMS	pI	MW (kDa)	Conserved domains	SL	Alternative names	Reference for alternative names
*HvHAK1*	HORVU.MOREX.r2.1HG0007070.1	783	8	14	8.25	87.65	K_trans superfamily	PM		
*HvHAK2*	HORVU.MOREX.r2.1HG0054170.1	812	6	11	6.95	89.84	K_trans superfamily	PM		
*HvHAK3*	HORVU.MOREX.r2.1HG0054790.1	736	4	11	8.58	80.97	kup	PM		
*HvHAK4*	HORVU.MOREX.r2.2HG0092260.1	780	6	11	8.25	86.84	K_trans superfamily	PM		
*HvHAK5*	HORVU.MOREX.r2.2HG0093910.1	777	8	13	8.78	86.71	K_trans superfamily	PM	*HvHAK2*	[16]
*HvHAK6*	HORVU.MOREX.r2.2HG0117130.1	853	9	12	5.83	94.30	PLN00151	PM		
*HvHAK7*	HORVU.MOREX.r2.2HG0130150.1	875	2	12	8.87	98.04	K_trans superfamily	PM		
*HvHAK8*	HORVU.MOREX.r2.2HG0135750.1	775	8	12	8.95	86.41	kup	PM	*HvHAK1; HvHAK1A*	[16, 50]
*HvHAK9*	HORVU.MOREX.r2.2HG0135780.1	775	8	10	6.59	86.61	kup	PM		
*HvHAK10*	HORVU.MOREX.r2.2HG0158090.1	792	8	14	8.59	88.73	K_trans superfamily	PM		
*HvHAK11*	HORVU.MOREX.r2.2HG0158800.1	857	9	13	5.65	95.34	K_trans superfamily	PM		
*HvHAK12*	HORVU.MOREX.r2.3HG0261230.1	784	8	12	8.49	86.86	K_trans superfamily	PM		
*HvHAK13*	HORVU.MOREX.r2.3HG0261730.1	740	5	11	8.89	81.51	K_trans	PM		
*HvHAK14*	HORVU.MOREX.r2.3HG0262540.1	785	8	13	8.96	87.66	K_trans superfamily	PM	*HvHAK4*	[50]
*HvHAK15*	HORVU.MOREX.r2.3HG0265630.1	743	6	12	8.75	82.20	kup	PM		
*HvHAK16*	HORVU.MOREX.r2.4HG0314340.1	787	8	12	6.80	87.50	K_trans superfamily	PM		
*HvHAK17*	HORVU.MOREX.r2.4HG0339220.1	770	8	11	8.86	85.72	kup	PM	*HvHAK1B*	[50]
*HvHAK18*	HORVU.MOREX.r2.5HG0395650.1	866	8	12	6.04	95.99	PLN00151	PM	*HvHAK3*	[50]
*HvHAK19*	HORVU.MOREX.r2.5HG0402150.1	724	7	11	8.71	79.93	K_trans	PM		
*HvHAK20*	HORVU.MOREX.r2.5HG0420460.1	785	8	14	8.16	87.86	K_trans superfamily	PM		
*HvHAK21*	HORVU.MOREX.r2.6HG0455930.1	736	3	11	8.27	80.97	kup	PM		
*HvHAK22*	HORVU.MOREX.r2.6HG0507640.1	769	8	13	8.30	85.03	K_trans superfamily	PM		
*HvHAK23*	HORVU.MOREX.r2.6HG0508040.1	800	9	11	8.86	90.60	K_trans superfamily	PM		
*HvHAK24*	HORVU.MOREX.r2.7HG0557110.1	772	9	13	7.82	85.49	K_trans superfamily	PM		
*HvHAK25*	HORVU.MOREX.r2.7HG0564560.1	724	7	12	8.99	80.93	K_trans	PM		
*HvHAK26*	HORVU.MOREX.r2.7HG0599600.1	830	8	12	8.70	91.29	K_trans superfamily	PM		
*HvHAK27*	HORVU.MOREX.r2.7HG0610980.1	761	8	11	8.63	84.24	K_trans superfamily	PM		

*aa* Amino acids, *pI* Isoelectric point, *MW* Molecular weight, *SL* Subcellular localization, *TMS* Transmembrane segment, *PM* Plasma membrane

### Phylogenetic analysis of HvHAK transporters

**Fig. 1:**
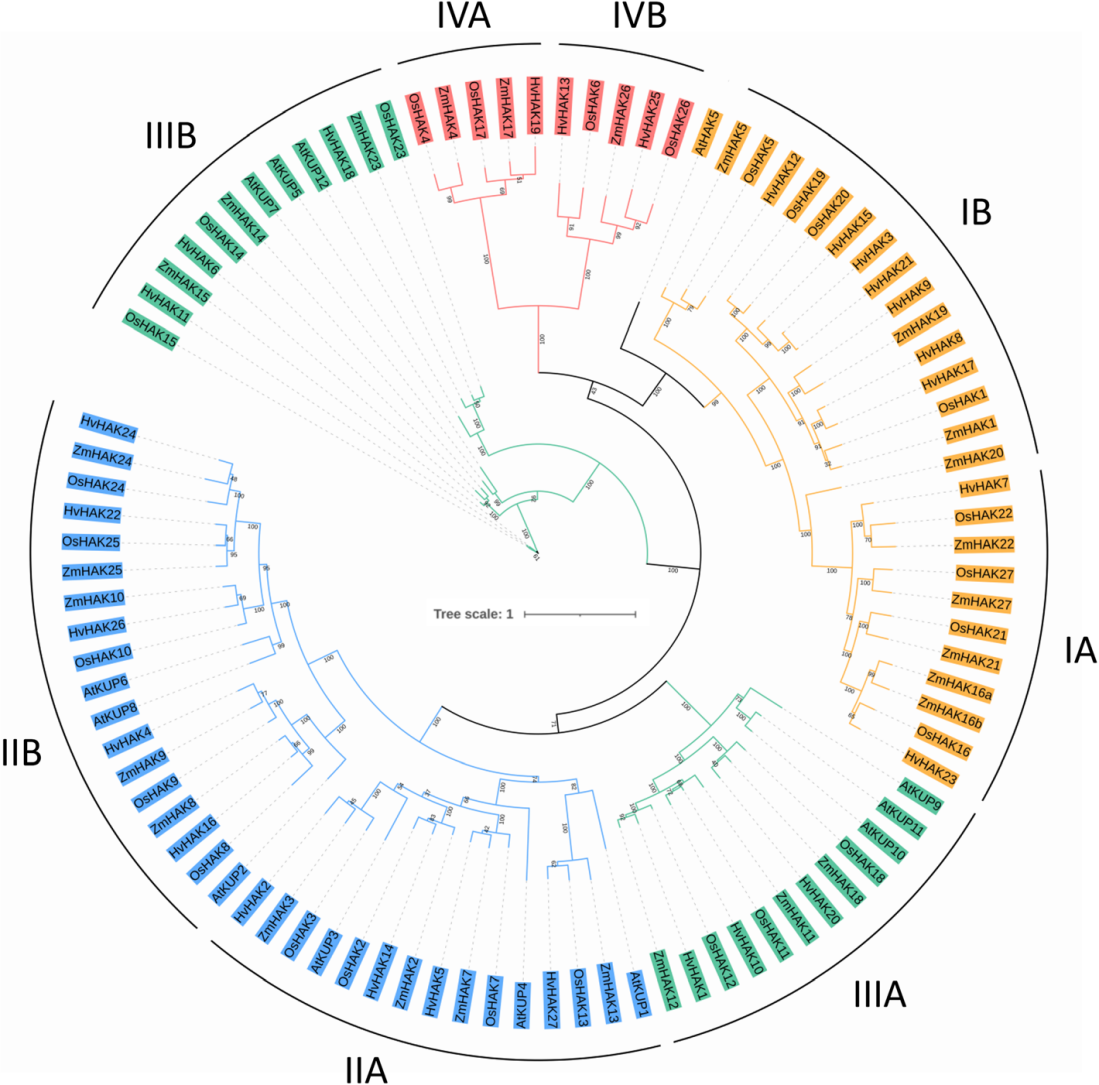
Phylogenetic tree of HAK/KUP/KT family proteins from barley, rice, maize and *Arabidopsis*. The HAK/KUP/KT family proteins were divided into four clusters and were indicated with different colors. Yellow, blue, green and red clusters represented cluster I, II, III and IV, respectively. Hv, *Hordeum vulgare*; Os, *Oryza sativa*; Zm, *Zea mays*; At, *Arabidopsis thaliana*

The protein sequences of 13 AtKUPs in *Arabidopsis* [51], 27 OsHAKs in rice [24], 27 ZmHAKs in maize [26], and together with 27 HvHAKs in barley, were aligned with MAFFT (https://www.ebi.ac.uk/Tools/msa/mafft/) and used to construct phylogenetic tree by maximum-likelihood (ML) method (Fig. 1). Based on the classification criteria of HAK transporters in *Arabidopsis*, rice and maize, the identified 27 barley HvHAKs could be classified into four clusters, and each of them could be further subdivided into sub-cluster A and B (Fig. 1). Clusters I and II each contained nine transporters, with IA, IB, IIA and IIB each containing two (HvHAK7 and 23), seven (HvHAK3, 8, 9, 12, 15, 17 and 21), four (HvHAK2, 5, 14 and 27) and five members (HvHAK4, 16, 22, 24 and 26), respectively (Fig. 1). Cluster III consisted of six transporters, with three members in both IIIA (HvHAK1, 10 and 20) and IIIB (HvHAK6, 11 and 18) (Fig. 1). Only three HvHAK transporters were categorized into cluster IV, with one member (HvHAK19) in IVA and two members (HvHAK13 and 25) in IVB (Fig. 1).

### Motif, domain and gene structure analyses of *HvHAKs*

**Fig. 2:**
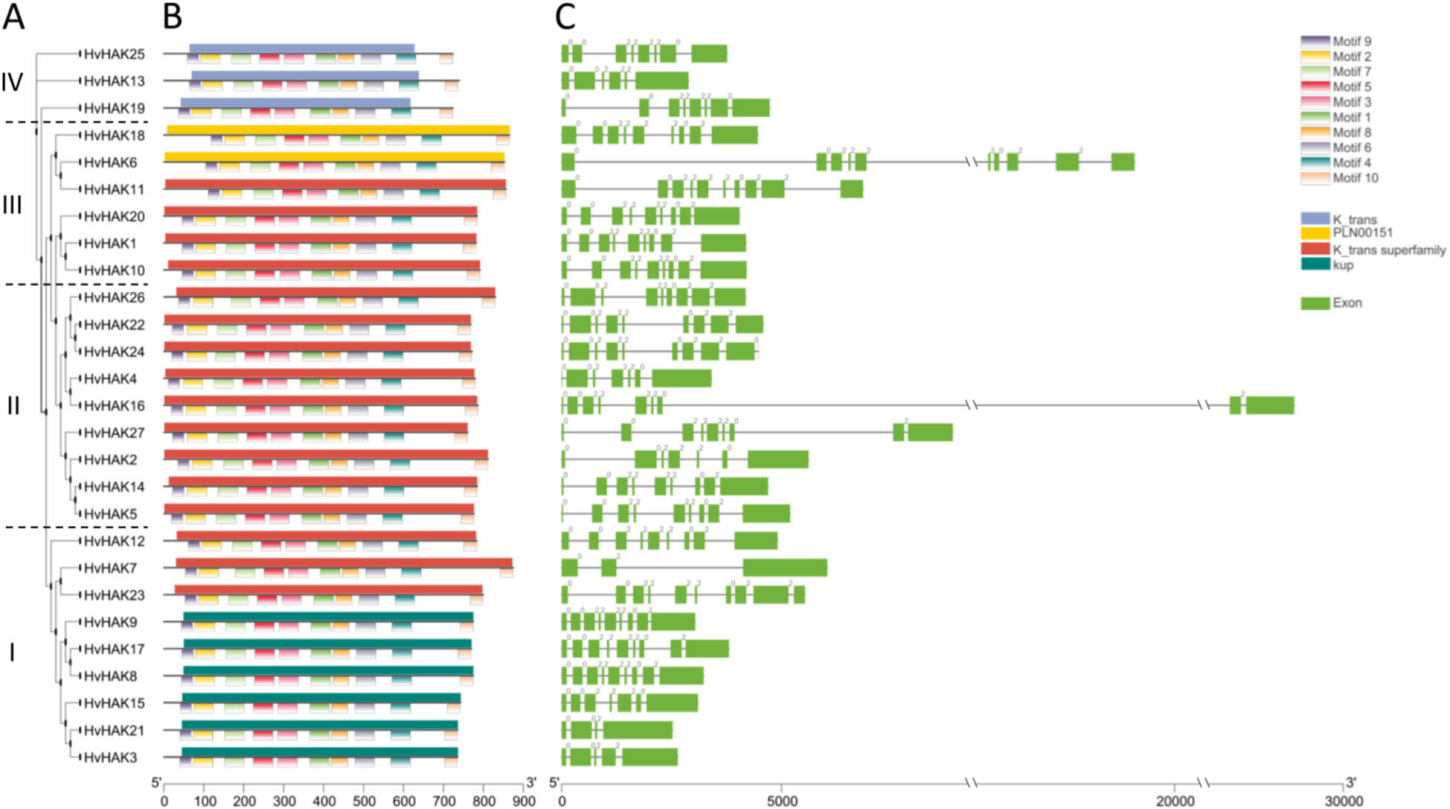
Phylogenetic relationship and sequence characteristics of HvHAK proteins. **a** Phylogenetic tree of HvHAK proteins. **b** Conserved motifs and domains of HvHAK proteins. Conserved domains and motifs were indicated on the upper side and lower side of protein sequences, respectively. **c** Structure of *HvHAK* genes. Introns and exons were represented with black lines and green boxes, respectively

The conserved motifs and domains of HvHAK protein sequence and structures of *HvHAK* genes were further analyzed (Fig. 2). Phylogenetic analysis confirmed the classification of 27 HvHAKs (Figs. 1 and 2a). Ten conserved motifs were identified in all 27 HvHAK sequences (Fig. 2b). According to NCBI conserved domain database, there are eight types of members for “K_trans superfamily” (cl15781), and three of them were identified in HvHAK protein sequences (Fig. 2b). The conserved domains of three, two and six HvHAK proteins were specifically identified as “K_trans”, “PLN00151” and “kup”, respectively, while those of the other 16 HvHAK sequences were identified as “K_trans superfamily” because of relatively low specificity (Fig. 2b). Cluster I and III contained “kup” and “K_trans superfamily”, “PLN00151” and “K_trans superfamily”, respectively; while cluster II and IV were solely comprised of “K_trans superfamily” and “K_trans”, respectively (Fig. 2b). Moreover, gene structures of barley *HvHAKs* were highly variable, containing 3–10 exons and 2–9 introns (Table 1; Fig. 2c).

Totally there were 14 (51.9%) *HvHAK* genes which each contained eight introns, and there were 4, 3 and 2 genes which contained nine, six and seven introns, respectively; and other 4 genes possessed five, four, three and two introns, respectively (Fig. 2c). Intron numbers also differed among the different clusters (Fig. 2c); cluster I was the most variable, with six genes each containing two, three, four, six, eight and nine introns, respectively; three genes in cluster II had six, eight and nine introns, respectively; and two genes in both cluster III and IV had eight and nine, five and seven introns, respectively (Fig. 2c). These introns could be classified into three types based on phases: phase 0, phase 1 and phase 2 [52]. Interestingly, phases 0 and 2 introns were detected, while phase 1 introns were not found in barley *HvHAK* genes (Fig. 2c). The phylogenetically most closely related members are prone to have the similar exon/intron structure. For instance, gene pairs *HvHAK6* and *HvHAK11*, *HvHAK1* and *HvHAK10*, *HvHAK5* and *HvHAK14*, and *HvHAK8* and *HvHAK17* contained the same exon/intron numbers, respectively, and the exon configuration of each gene pair was almost the same regardless of the difference in intron length (Fig. 2c). However, the gene pairs of *HvHAK4* and *HvHAK16*, and *HvHAK7* and *HvHAK23* showed the difference in both intron number and exon configuration (Fig. 2c).

### Identification of *cis*-acting elements in *HvHAK* genes

**Fig. 3:**
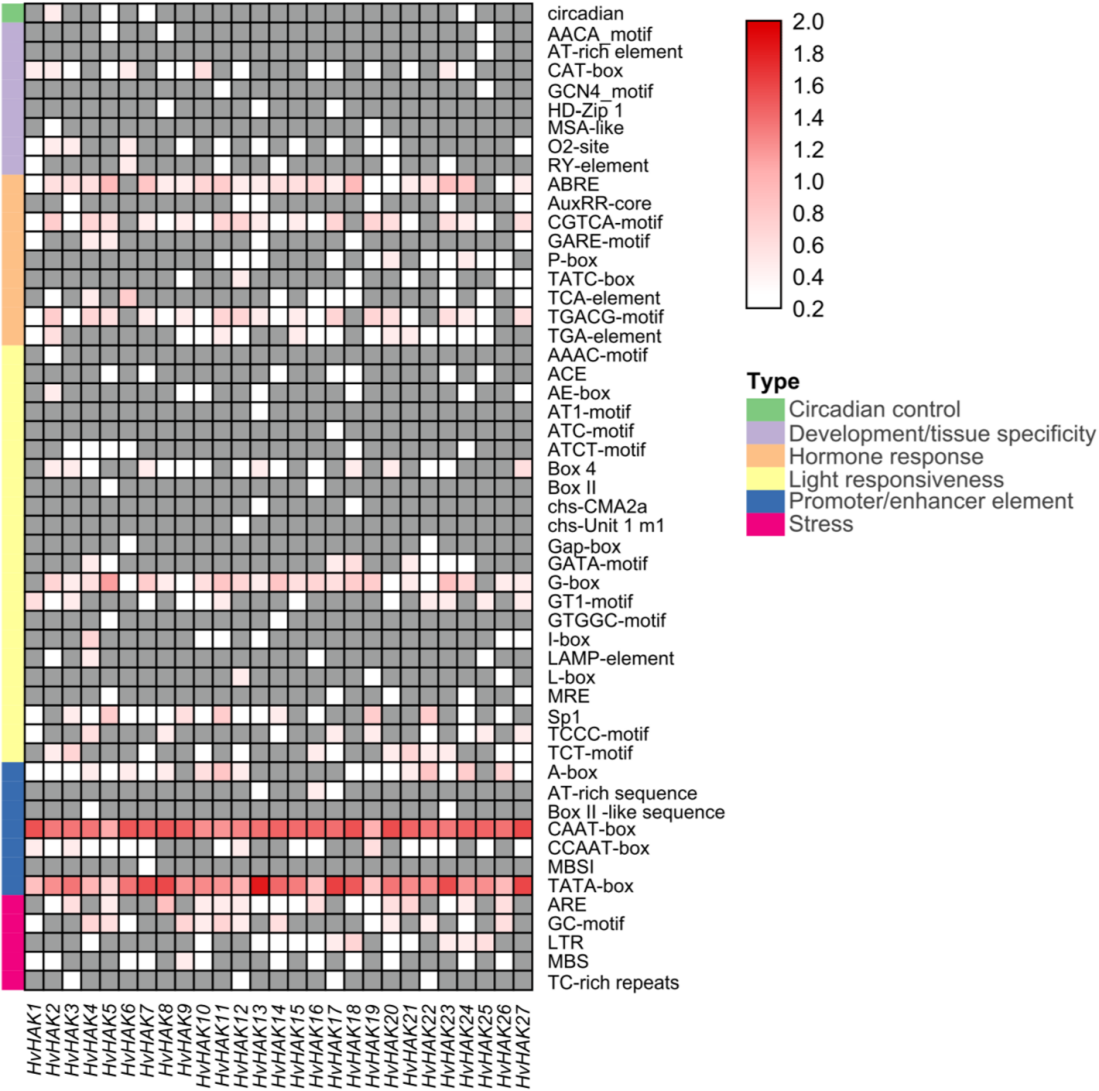
Analysis of *cis*-acting elements in *HvHAKs*. Prediction of *cis*-acting elements was conducted on 2 kb sequences upstream of coding sequences of *HvHAKs*. Quantity of each kind of *cis*-acting elements were normalized by log _10_(number + 1) and then used for heatmap construction. All *cis*-acting elements could be divided into six types

A 2 kb sequence in the upstream of *HvHAK* coding sequences was used for identification of *cis*-acting element. In total, 52 cis-acting elements were identified, and categorized into 6 types according to their functional annotations (Fig. 3; Additional file 3). Light responsiveness-related elements were the most abundant (22, 42.3%), followed by hormone response-related (9, 17.3%), development/tissue specificity-related (8, 15.4%), promoter/enhancer element-related (7, 13.5%) and stress-related (5, 9.6%) ones (Fig. 3). Only one kind of elements was identified as circadian control (Fig. 3). CAAT-box and TATA-box from promoter/enhancer element, which are binding sites for RNA polymerase and responsible for transcription efficiency, were ubiquitously identified in all *HvHAKs*, indicating that they might play the pivotal roles in controlling the initiation and level of *HvHAKs* expression. Moreover, ABRE, as well as CGTCA-motif and TGACG-motif (from hormone response type), which are involved in abscisic acid (ABA) and methyl jasmonate (MeJA) responsiveness, respectively, were widely present in *HvHAKs* (Fig. 3). G-box in light responsiveness was also extensively distributed in promoter regions of *HvHAKs* (Fig. 3). These results suggest that *HvHAKs* might play critical roles in phytohormone metabolism and environmental responses.

### Synteny analysis of *HvHAKs* in four monocotyledons

**Fig. 4:**
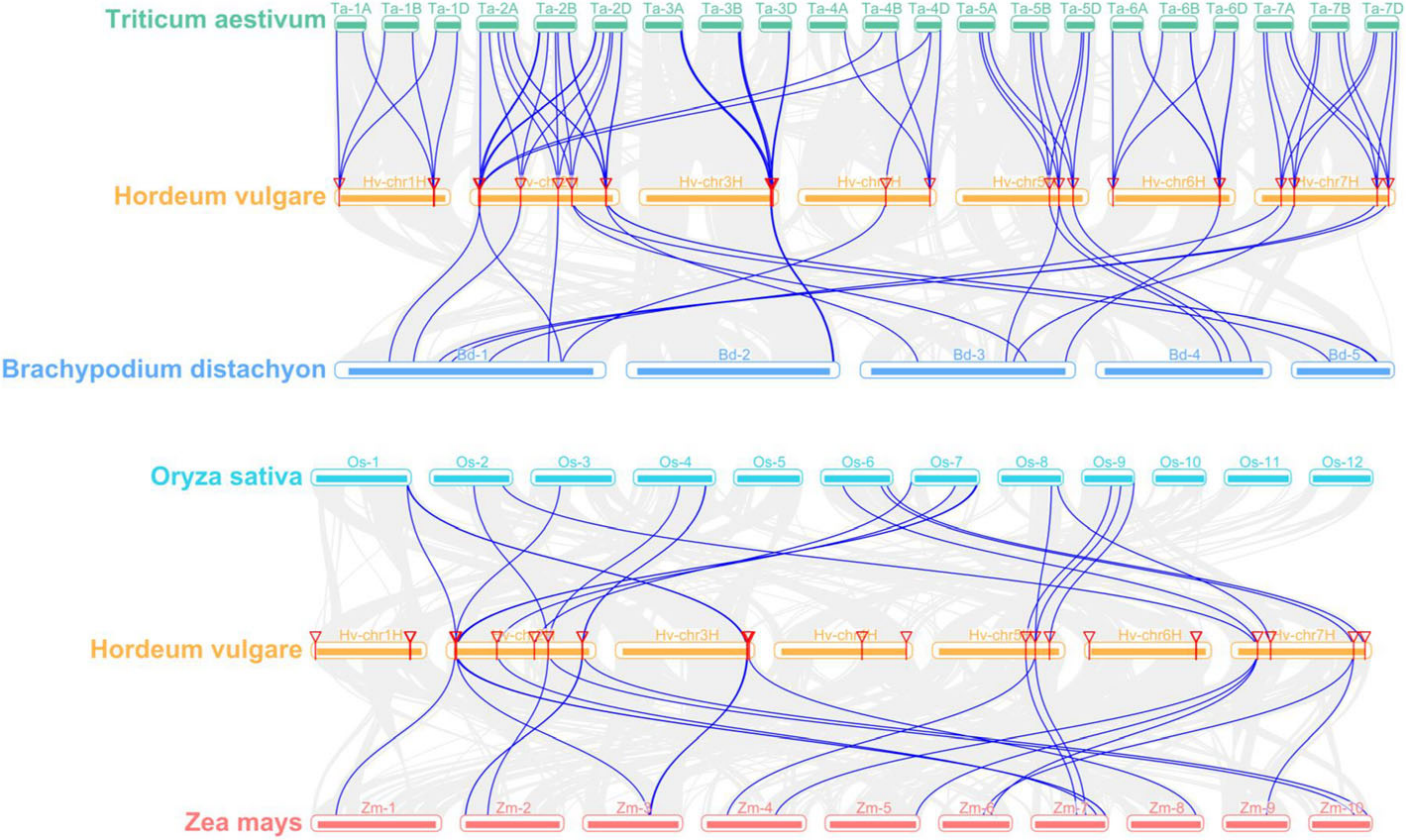
Synteny analyses between *HAK* genes of barley and four monocotyledonous species (*Triticum aestivum*, *Brachypodium distachyon*, *Oryza sativa* and *Zea mays***).** *HvHAK* genes are anchored on barley choromosomes. Grey lines indicated collinear blocks between barley and other plant genomes, while blue lines highlighted syntenic *HAK* gene pairs

The syntenic relationships of *HvHAKs* in four monocotyledonous plants (*Triticum aestivum*, *Brachypodium distachyon*, *Oryza sativa* and *Zea mays*) were investigated (Fig. 4; Additional file 4). Totally 73 pairs of orthologous genes were identified between barley and wheat, comprising 25 (92.6%; except *HvHAK3* and *HvHAK9*) *HvHAKs* and 72 *TaHAKs* (Fig. 4). Twenty-two pairs of orthologous genes were observed between barley and *Brachypodium distachyon*, barley and rice, and barley and maize, respectively, including 19 (70.4%) *HvHAKs* and 21 *BdHAKs*, 17 (63.0%) *HvHAKs* and 21 *OsHAKs*, 13 (48.1%) *HvHAKs* and 20 *ZmHAKs*, respectively (Fig. 4). Orthologous genes of six barley *HvHAKs* (*HvHAK1*, *2*, *15*, *17*, *21* and *23*) were not detected in *Brachypodium distachyon*, rice and maize genomes, although found in wheat genome. Totally, 22 *HvHAKs* each had three orthologous genes in wheat, while only *HvHAK24* had three orthologous genes in maize. Three *HvHAKs*, five *HvHAKs* and seven *HvHAKs* each had two orthologous genes in *Brachypodium distachyon*, rice and maize genomes, respectively. One *TaHAK* and one *BdHAK* were orthologous to two *HvHAKs* (*HvHAK4* and *HvHAK16*), and one *OsHAK* and one *ZmHAK* were orthologous to the other two *HvHAKs* (*HvHAK5* and *HvHAK14*). Besides, another *ZmHAK* was also orthologous to *HvHAK12* and *HvHAK13*. These results indicate that *HvHAKs* in barley are phylogenetically closer to *TaHAKs* in wheat than to *BdHAKs* in *Brachypodium distachyon*, *OsHAKs* in rice and *ZmHAKs* in maize.

### Tissue expression patterns of *HvHAKs*

**Fig. 5:**
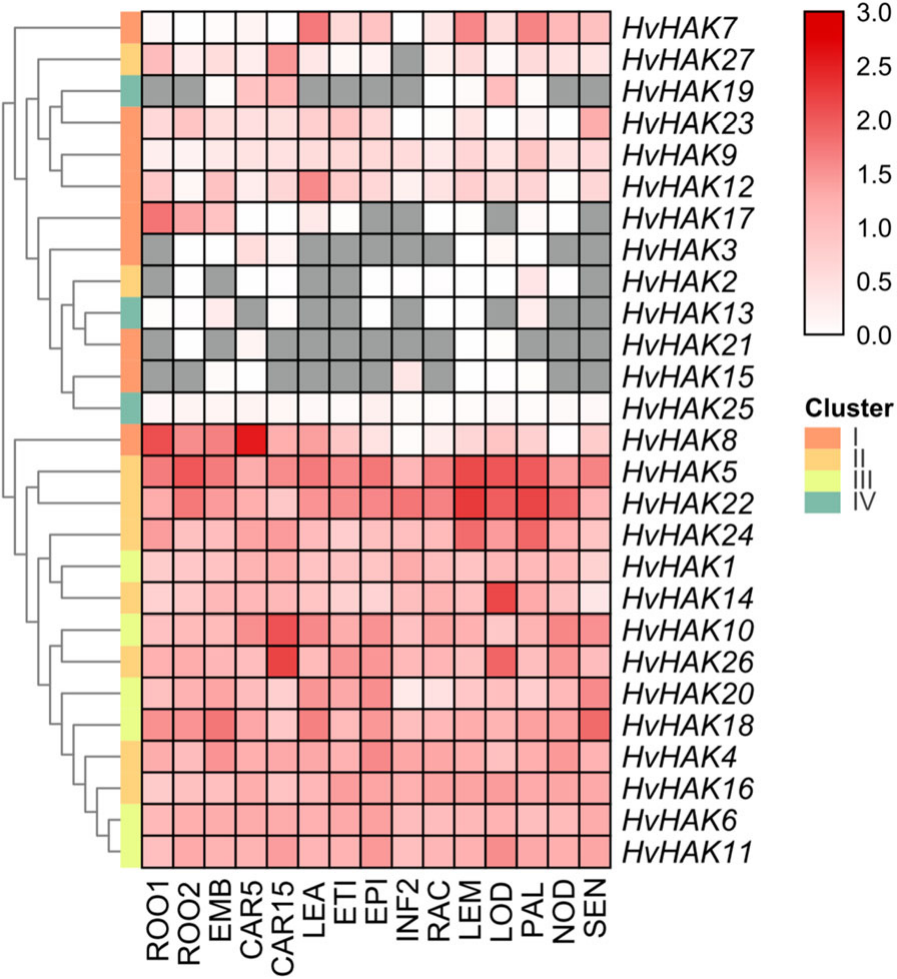
Expression profiling of *HvHAKs* in 15 tissues based on transcriptomic data. FPKM values were normalized by log_10_(FPKM+ 1) transformation. ROO1, roots from seedlings (10 cm shoot stage); ROO2, roots (28 DAP); EMB, 4 day embryos; CAR5, developing grain (5 DAP); CAR15, developing grain (15 DAP); LEA, shoots from seedlings (10 cm shoot stage); ETI, etiolated seedling, dark condition (10 DAP); EPI, epidermal strips (28 DAP); INF, developing inflorescences (1–1.5 cm); RAC, inflorescences, rachis (35 DAP); LEM, inflorescences, lemma (42 DAP); LOD, inflorescences, lodicule (42 DAP); PAL, dissected inflorescences, palea (42 DAP); NOD, developing tillers, 3rd internode (42 DAP); SEN, senescing leaves (56 DAP)

Tissue expression patterns of *HvHAKs* were investigated in 15 tissues based on transcriptomic data from Barlex database (Additional file 5). Hierarchical cluster analysis revealed that 27 *HvHAKs* could be divided into two groups according to their expression levels (Fig. 5). One group comprised 14 *HvHAKs*, and of them 13 genes were from cluster II and cluster III, with only one gene (*HvHAK8*) from cluster I (Fig. 5). Genes in this group were expressed ubiquitously in all 15 tissues with relatively high levels. The other group consisted of 13 genes, with eight from cluster I, two from cluster II and three genes from cluster IV (Fig. 5). The expression levels of *HvHAKs* in this group were comparatively lower than that of the genes belonging to the former group, with eight genes being not detected in certain specific tissues (Fig. 5). Moreover, the expression of *HvHAK2*, *HvHAK3* and *HvHAK21* was undetectable in seedling roots, while detected in the roots at 28 d after heading (Fig. 5). Furthermore, *HvHAK13* was not expressed, and *HvHAK15* and *HvHAK21* were mildly expressed in developing grains (5 d after heading). However, *HvHAK13* was mildly expressed, but *HvHAK15* and *HvHAK21* were not expressed at 15 d after heading (Fig. 5). These results indicate that the expression of *HvHAKs* is not only tissue-dependent, but also varies with development phase.

### Expression patterns of *HvHAKs* responding to abiotic stresses

The effects of salt stress (200 mM NaCl), drought stress (20% PEG8000) and potassium deficiency (0.01 mM K^+^) on barley growth were investigated. All three abiotic stress treatments inhibited shoot growth of barley seedlings, and resulted in abnormal root phenotypes (Additional file 6).

**Fig. 6:**
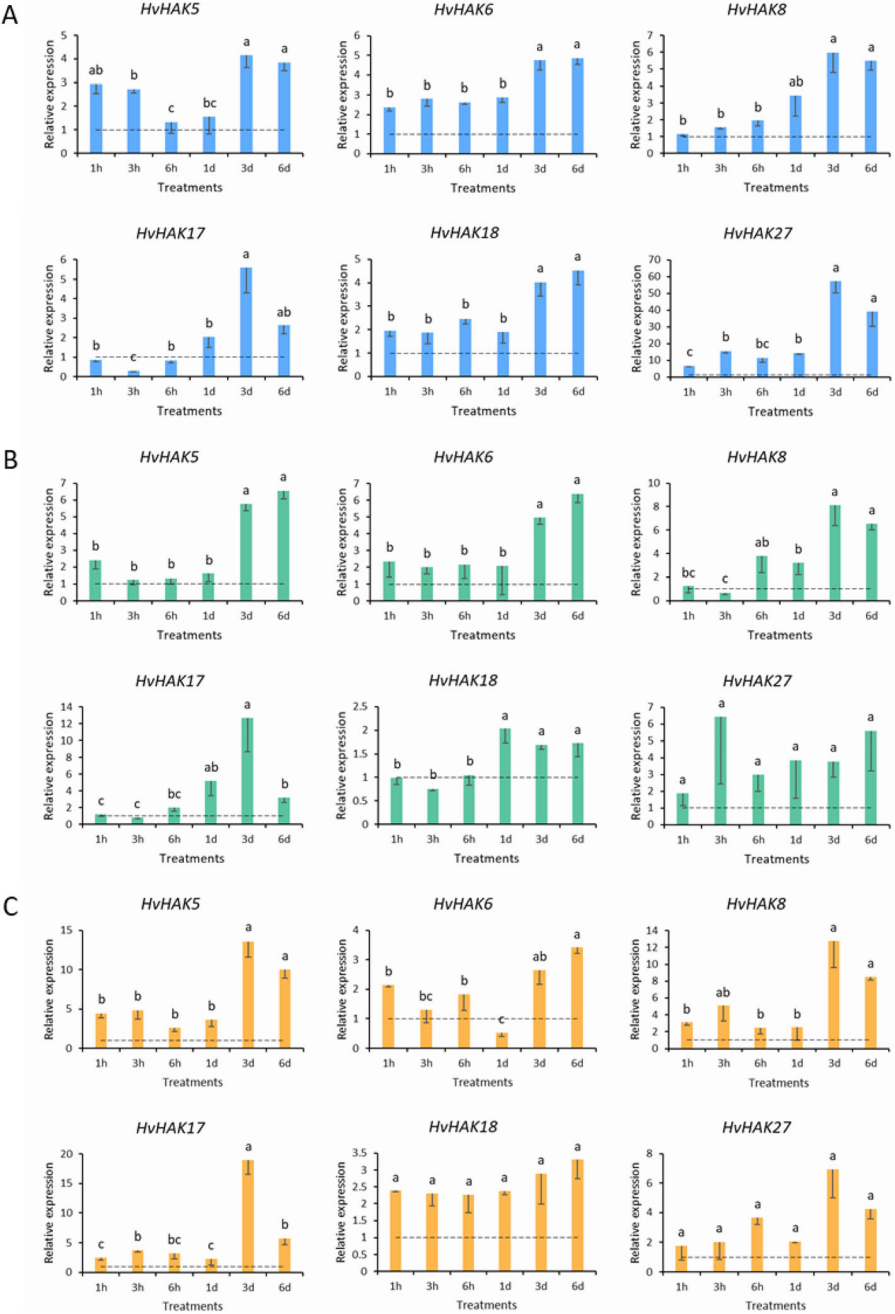
Expression profiling of six *HvHAK* genes in response to abiotic stresses at seedling stage. **a** Expression of *HvHAKs* in response to salt stress (200 mM NaCl). **b** Expression of *HvHAKs* in response to osmotic stress (20% PEG8000). **c** Expression of *HvHAKs* in response to potassium deficiency (0.01 mM K^+^). Dotted lines indicated the expression levels of *HvHAKs* in control seedlings. Lowercase letters indicated the significant difference at *p* < 0.05

Under salt stress, all *HvHAKs* examined were up-regulated, but their expression patterns differed (Fig. 6). *HvHAK5*, *HvHAK6*, *HvHAK18* and *HvHAK27* were rapidly induced after one-hour salt treatment, and displayed continuous up-regulation, while *HvHAK8* was not induced until salt treatment for 3 h (Fig. 6a). Besides, *HvHAK17* was first down-regulated after treatment (1–6 h), and was up-regulated after one-day salt treatment (Fig. 6a). In addition, *HvHAK6*, *HvHAK18* and *HvHAK27* showed significantly higher up-regulation after long-term (3–6 d) salt treatment than after short-term (1 h to 1 d) treatment (Fig. 6a).

Under osmotic stress, *HvHAK5*, *HvHAK6,* and *HvHAK27* were continuously induced during the whole experiment period (Fig. 6b). However, *HvHAK8*, *HvHAK17* and *HvHAK18* remained little change after one-hour treatment and even down-regulated after three-hour treatment, while they were up-regulated after treatment for 6 h and 1 d (Fig. 6b). Similarly, *HvHAK5*, *HvHAK6* and *HvHAK8* had significantly higher expression after long-term (3 d and 6 d) treatment than after short-term (< 1 d) treatment (Fig. 6b).

All *HvHAK* genes examined were up-regulated under K deficiency, except *HvHAK6*, which was down-regulated at 1 d after treatment (Fig. 6c). Besides, the expression of *HvHAK18* remained relatively stable during the whole period of treatment (Fig. 6c).

## Discussion

So far, the HAK/KUP/KT potassium transporter family has been identified and analyzed in many plant species, including *Arabidopsis* [50, 51], rice [23, 24, 43], wheat [25], maize [26] and cassava [44]. In the current study, we identified 27 *HvHAK* genes in barley (Table 1) using data of barley genome [48] and the improved annotated reference genome assembly [49], and then investigated their phylogenetic relationships, sequence characteristics, *cis*-acting elements, syntenic relationships, tissue expression patterns and expression profiling in response to abiotic stress.

### Nomenclature and classification of *HvHAKs*

*HAK* genes in maize and wheat were named according to their phylogenetic relationships with *OsHAKs* [25, 26]. The first identified *HAK* gene in barley was named as *HvHAK1* due to its high homology to *Escherichia coli Kup* and *Schwanniomyces occidentalis HAK1*, and another gene with only partial length (incomplete at 5′ end) was named as *HvHAK2* [16]. Rubio et al. [50] isolated three new cDNAs of *HAK* genes in barley, and found one of them had more than 90% similarity to *HvHAK1*. Consequently, they named it as *HvHAK1B* (*HvHAK1* was designated as *HvHAK1A*), and the other two genes as *HvHAK3* and *HvHAK4*, respectively. *HvHAK1*/*HvHAK1A* and *HvHAK1B* were phylogenetically most closely related to *OsHAK1*, while *HvHAK2*, *HvHAK3* and *HvHAK4* showed the closest phylogenetic relationships with *OsHAK7*, *OsHAK23* and *OsHAK2*, respectively (Table 1; Fig. 1). Therefore, naming *HvHAKs* based on their phylogenetic relationships with *OsHAKs* seems not to be straightforward and understandable. Thus, 27 *HvHAKs* were designated as *HvHAK1* to *HvHAK27* according to their chromosomal locations (Table 1).

*OsHAKs* were divided into four clusters on the basis of phylogenetic analysis, with cluster I, II, II and IV having eight, nine, six and four genes, respectively [24]. Barley *HAK* genes can be also classified into four clusters according to the classification criteria of *OsHAKs* in rice, with cluster I, II, III and IV having nine, nine, six and three genes, respectively (Fig. 1). Notably, *OsHAK13* was subdivided into cluster IIB previously [24], while it was subdivided into cluster IIA in this study (Fig. 1). This inconsistency might be caused by the difference in the methods of protein sequence alignment and phylogenetic construction. Gupta et al. [24] adopted ClustalX in the protein sequence alignment of OsHAKs, while MAFFT was used in this study, which was widely recommended for its higher accuracy in multiple sequence alignment [53]. In addition, neighbor joining (NJ) was used in the phylogenetic construction of *OsHAKs* [24]. NJ method is widely used due to its high computational efficiency, however, it cannot account for the high variances of large distance estimates and is also sensitive to the gaps in the sequence alignment [54]. Maximum likelihood, which was adopted in this study, has an obvious advantage over NJ method in understanding the sequence evolution process [54].

### Similarities and differences of *HAK* genes between barley and other plants

In this study, 27 *HAK* genes were identified in barley, basically similar to those found in rice [24], maize [26] and *Brachypodium distachyon* [55], although these plants show a huge difference in genome sizes, ranging from ~ 355 Mb of *Brachypodium distachyon* to ~ 5.3 Gb of barley. In addition, the chromosome distribution of 27 *HvHAK* genes are uneven. Similarly, *ZmHAKs* were unevenly distributed on 10 chromosomes [26]. However, the chromosome distribution of *OsHAKs* are almost even [24]. The duplication events (including segmental duplication and tandem duplication) could be observed in both *OsHAKs* and *ZmHAKs* [26, 43], but not detected in *HvHAKs* (Additional file 2). The length in amino acid sequences of HvHAKs, OsHAKs and ZmHAKs were similar, ranging from 724 to 875, 697 to 877 and 642 to 921 aa, respectively (Table 1) [24, 26]. Introns of *HvHAK* genes varied from 2 to 9, being similar to those of *ZmHAK* and *OsHAK* genes, with an exception of *OsHAK22*, which had only one intron. Interestingly, the *HAK* genes with 8 introns were predominant in barley (14, 51.9%), rice (12, 44.4%) and maize (16, 59.3%) [24, 26]. Intron phase determines whether exons are targeted for alternative splicing. Generally, exons flanked by same-phase introns are possible for alternative splicing, while those flanked by the different-phase introns cannot be alternatively spliced [52]. On the whole the most introns of *HAK* genes in rice and maize belonged to phase 0 and phase 1, with only four and three introns in phase 2 for *OsHAKs* and *ZmHAKs*, respectively [24, 26]. However, introns of *HvHAKs* all belong to phase 0 or phase 2, and no introns were observed at phase 1 (Fig. 2), suggesting that *HvHAKs* had the different expression over the phase from *OsHAKs* and *ZmHAKs*. The numbers of transmembrane segments (TMS) for OsHAKs, ZmHAKs and HvHAKs were in the range of 11–15, 9–14, and 10–14, respectively (Table 1) [24, 26]. The *HAK* genes with 13 TMS (11, 40.7%) and 11 TMS (9, 33.3%) were dominant in *OsHAKs* and *ZmHAKs*, while those with 11 and 12 TMS (18, 66.7%) were the most abundant in *HvHAKs* (Table 1) [24, 26].

All HAK proteins in rice, maize and wheat contain a conserved domain, “K_trans” (PF02705) [24–26]. In the present study, a conserved domain of “K_trans superfamily” (cl15781) was also identified in all HvHAK transporters (Fig. 2b). In addition, 1 conserved motifs were detected, and they were almost evenly distributed along HvHAK sequences, similar to motifs in TaHAKs [25]. For the *cis*-acting elements related to light responsiveness, they were the most abundant in *HvHAKs*, while quite fewer in *OsHAKs* and *ZmHAKs* [24, 26]. On the other hand, Ca^2+^-responsive elements could be detected in the most *OsHAKs* and *ZmHAKs*, but not observed in *HvHAKs* (Fig. 3). It may be concluded that there is a great difference in *cis*-acting elements among *OsHAKs*, *ZmHAKs* and *HvHAKs*.

Based on the classification criteria of *HAK* genes in rice and maize, *HvHAKs* can be also divided into four clusters (Fig. 1). Synteny analysis identified 73 *HAK* orthologous gene pairs (comprising 25 *HvHAKs* and 72 *TaHAKs*) in barley and wheat genomes, much more than those between barley and *Brachypodium distachyon* genomes, and between rice and maize genomes, respectively (Fig. 4; Additional file 4). Interestingly, each of the 22 *HvHAK* genes was orthologous to three *TaHAK* genes, which might be attributed to the fact that wheat is an allohexaploid composed of three distinct ancestral genomes, viz. A, B and D [56]. If this explanation is reasonable, the phenomena that *HvHAK15* and *HvHAK16* were orthologous to two (lacking orthologous gene on genome D) and one *TaHAKs* (lacking orthologous genes on genomes A and B), respectively, can be described to gene loss events during evolution (Additional file 4). In addition, *HvHAK4* was orthologous to four *TaHAK* genes on two chromosomes, suggesting that both gene loss and genomic recombination might have occurred (Additional file 4). Notably, according to the synteny analysis in this study, at least 72 *HAK* genes should be present in wheat genome, while only 56 *TaHAK* genes were identified in a previous research [25]. The inconsistency can be attributed to the imperfect assembly and annotation of wheat genome, as well as the workflow of gene family identification. Based on the barley genome assembly released in 2017 [48], only 24 *HAK* genes were identified in barley genome (three genes with incomplete K^+^ transporter domains were arbitrarily discarded), while using the recently released barley reference genome assembly [49], we identified 27 *HvHAK* genes. On the other hand, 22 *HAK* orthologous gene pairs were identified between barley and other three plants examined in this study (Fig. 4; Additional file 4). In addition, 19, 17 and 13 *HvHAKs* were orthologous to 21 *BdHAKs*, 21 *OsHAKs* and 20 *ZmHAKs*, respectively, being consistent with the phylogenetic relationships between barley and these plants (Fig. 4; Additional file 4). No orthologous gene was found for *HvHAK3* and *HvHAK9* in other three genomes, indicating that these two genes might originate from ectopic duplication of *HvHAK2* and *HvHAK8*, respectively (Fig. 1) [57].

### Expression of *HvHAKs* in different tissues and in response to abiotic stresses

Analysis of tissue expression pattern revealed that *HvHAK* genes could be divided into two groups (Fig. 5). One group, consisting of 14 genes, was constitutively and highly expressed in all 15 tissues, while another group, consisting of 13 genes, only expressed in the specific tissues with lower levels (Fig. 5). In the previous studies, expression of *HAK* genes in *Arabidopsis*, rice and wheat also showed the great difference in the tissues and levels [24, 25, 58].

Salt, osmotic (drought) and K deficiency stresses had the dramatic effect on the expression of the examined six *HvHAK* genes (Fig. 6). Maintaining efficient K^+^ uptake is prerequisite for K^+^/Na^+^ homeostasis and salt tolerance when plants are exposed to salt stress [59]. In this study, the expression of the six *HvHAKs* was up-regulated under salt stress (Fig. 6a), contributing to high K^+^/Na^+^ ratio and salt tolerance. The similar results were also observed for *OsHAKs* in rice and for *MeKUPs* in cassava [44, 60]. It is well recognized that plants respond to drought stress via both ABA-dependent and ABA-independent pathways [61, 62]. It was reported that increasing cytosolic ion concentrations through absorbing and accumulating K^+^ is a relatively fast and cost-effective way for plants to enhance intracellular osmotic adjustment and drought tolerance [63, 64]. In the present study, the expression of *HvHAK* genes were up-regulated in a time-dependent manner when plants were exposed to osmotic stress (drought) (Fig. 6b), being consistent with the previous reports on *TaHAKs* in wheat and *MeKUPs* in cassava [25, 44]. In general, K^+^ transporter genes are up-regulated under K deficiency conditions [25, 60, 65]. In this study, all examined *HvHAK* genes were up-regulated under K deficiency treatment (Fig. 6c). It was reported that the expression of *OsHAK1* was induced by K deficiency or salt stress, leading to increased K^+^ uptake and K^+^/Na^+^ ratio in roots [29]. In this study, the homolog genes of *OsHAK1* in barley, *HvHAK8* (alternative name as *HvHAK1* or *HvHAK1A*) and *HvHAK17* (alternative name as *HvHAK1B*), were also up-regulated under K deficiency or salt stress (Table 1; Figs. 1 and 6). In addition, the expression level of *HvHAKs* varied dramatically with the time of stress exposure (Fig. 6). For example, *HvHAK5* was slightly up-regulated (1.2–2.4 folds) at 1 h to 1 d after osmotic stress, while it was up-regulated significantly (5.8–6.6 folds) at 3–6 d after the treatment (Fig. 6b). Moreover, three genes (*HvHAK2*, *3* and *21*) were highly expressed in roots at 28 d after heading, but remained little change in seedling roots (Fig. 5). The same was true for *HvHAK13*, *HvHAK15* and *HvHAK21* in the developing grains (5 DAP and 15 DAP) (Fig. 5). Obviously, the response patterns of *HvHAK* genes to abiotic stress are dual-phases or even multi-phases. Thus, the suitable time of sampling should be taken into consideration when expression patterns of these *HvHAK* genes are analyzed and compared.

## Conclusions

In the current study, 27 *HAK* genes (*HvHAKs*) were identified in barley. The expression of these *HvHAKs* vary with the plant tissues and the time of exposed abiotic stress. Although all *HvHAKs* could be induced by salt, drought and K deficiency stresses, their response patterns and magnitudes showed the great variation. The obtained results should be helpful for us to make the comprehensive understanding of *HAK* family in barley.

## Methods

### Identification of HAK gene family in barley

The amino acid sequences of *HAK/KUP/KT* genes in *Arabidopsis thaliana* [51] and *Oryza sativa* [24] were downloaded from TAIR (https://www.arabidopsis.org/) and RAP-DB (https://rapdb.dna.affrc.go.jp/), respectively. The protein sequences of *AtKUPs* and *OsHAKs* were employed as queries to search against barley genome database (Morex V2 assembly) [49]. A total of 43 genes were identified as *HAK* gene family candidates in barley genome. The putative *HvHAKs* were then verified using NCBI Conserved Domain Database (https://www.ncbi.nlm.nih.gov/Structure/bwrpsb/bwrpsb.cgi), SMART (http://smart.embl-heidelberg.de/) and Pfam (https://pfam.xfam.org/), and the sequences without complete potassium transporter domain were discarded. Finally, 27 genes were identified as *HAK* gene family members in barley.

### Physicochemical properties, subcellular localizations and transmembrane segments of HvHAKs

The theoretical molecular weights (MW) and isoelectric points (pI) of HvHAKs were calculated using ExPASy (https://web.expasy.org/compute_pi/). Subcellular localizations and trans-membrane segments of HvHAKs were predicted using BUSCA (http://busca.biocomp.unibo.it/) [66] and TMHMM (https://services.healthtech.dtu.dk/service.php?TMHMM-2.0), respectively.

### Phylogenetic and syntenic analysis

The protein sequences of AtKUPs [51], OsHAKs [24] and ZmHAKs [26] were downloaded from TAIR (https://www.arabidopsis.org/), RAP-DB (https://rapdb.dna.affrc.go.jp/index.html) and maizeGDB (https://www.maizegdb.org/), respectively. HAK protein sequences from Arabidposis, rice, maize and barley were aligned using MAFFT (https://www.ebi.ac.uk/Tools/msa/mafft/) [67], and the phylogenetic tree was constructed with maximum-likelihood (ML) method using PhyML 3.0 (http://www.atgc-montpellier.fr/phyml/) [68]. The genome sequences and annotations of wheat, *Brachypodium distachyon*, rice and maize were downloaded from EnsemblPlants (http://plants.ensembl.org/info/website/ftp/index.html), and their syntenic relations with barley were analyzed and visualized using TBtools [69].

### Sequence, chromosomal location and duplication analyses of *HvHAKs*

Conserved motifs of HvHAKs were identified using MEME program (http://meme-suite.org/tools/meme) [70] with following parameters: classic motif discovery mode, zero or one occurrence per sequence (zoops), motif number was set to 10. Conserved domains were analyzed using NCBI Conserved Domain Database (https://www.ncbi.nlm.nih.gov/Structure/bwrpsb/bwrpsb.cgi). Motifs and conserved domains of HvHAKs as well as structures and chromosomal locations of *HvHAKs* were visualized using TBtools [69]. Gene duplication events were analyzed using TBtools following parameters described by Tombuloglu [69, 71].

### *cis*-acting element analysis

The 2000 bp sequences in the upstream of coding sequences of *HvHAKs* were extracted for *cis*-acting element analysis using Plant CARE database (http://bioinformatics.psb.ugent.be/webtools/plantcare/html/).

### Tissue expression patterns of *HvHAKs*

Raw data (FPKM) were downloaded from BARLEX (https://apex.ipk-gatersleben.de/apex/f?p=284:46:::NO:RP:P46_GENE_CHOICE:3) and normalized by log_10_(FPKM+ 1) transform. Expression heatmap was drawn using TBtools [69].

### Plant materials, growth conditions and abiotic stress treatments

Barley (*Hordeum vulgare* cv. Morex. From Professor Rugen Xu’s lab, Yangzhou University) seeds were sterilized with 10% commercial NaClO for 15 min and rinsed with tap water for 30 min [64]. Sterilized seeds were germinated in basic salt medium (BSM, 0.5 mM KCl + 0.1 mM CaCl_2_) for 2 d, and then BSM was changed to one-fifth Hoagland solution for another 4 d in growth room with a photoperiod of 14/10 h, light intensity of 200 ± 25 μmol·m^− 2^·s^− 1^, temperature of 23/18 °C (day/night) and relative humidity of 60%. Barley seedlings were grown for 6 d and then subjected to salt stress (200 mM NaCl) [72], osmotic stress (20% PEG8000) [64] and potassium deficiency (0.01 mM K^+^) [73] in background of 1/5 Hoagland solution. The seedlings growing in one-fifth Hoagland solution were set as control. The solutions were renewed every 2 days. After treatments for 1 h, 3 h, 6 h, 1 d, 3 d, and 6 d, roots of barley seedlings under both control and abiotic stress conditions were sampled for RNA extraction and qRT-PCR. All samples were collected in three replicates.

### qRT-PCR

Total RNA was extracted using MiniBEST Plant RNA Extraction Kit (9769, TaKaRa, Japan) following the manufacturer’s instructions. The cDNA was synthesized from total RNA (1 μg) using PrimeScript RT Master Mix (RR036A, TaKaRa, Japan) and was used as templates for qRT-PCR amplification. qRT-PCR amplification was performed with LightCycler 480 II (Roche, Basel, Switzerland) using iTaq Universal SYBR Green Supermix (1,725,124, Bio-Rad, USA). The relative gene expression was calculated based on the 2^−△△CT^ method using *actin* as the internal standard [74]. The primer sequences were listed in the Additional file 7.

## Supplementary Information

**Additional file 1.** Protein sequences of 27 HvHAKs.

**Additional file 2 **Chromosomal distribution of *HvHAK* genes. Chromosome names are displayed on the left side of chromosomes. *HvHAK* gene names are indicated on the right side of chromosomes.

**Additional file 3 ***cis*-acting elements in *HvHAK* genes.

**Additional file 4.** Orthologous gene pairs in synteny analysis.

**Additional file 5 **Tissue expression patterns of *HvHAKs*.

**Additional file 6.** Phenotypes of barley seedlings after abiotic stress treatments for 6 d.

**Additional file 7.** Primers for qRT-PCR.

## Data Availability

The datasets supporting the conclusions of this article are included within the article and its additional files.

## Abbreviations

ARF2: Auxin response factor 2
CHX: Cation/H^+^ exchanger
CPA: Monovalent cation/proton antiporter
HAK: High-affinity K^+^ transporter
HKT: High-affinity K^+^ transporter
KEA: K^+^ efflux antiporter
KT: K^+^ transporter
KUP: K^+^ uptake permease
MW: Molecular weight
NHX: Na^+^/H^+^ exchanger
NJ: Neighbor joining
NSCC: Non-selective cation channel
pI: Isoelectric point
SHY3: Short hypocotyl 3
TRH1: Tiny root hair 1
