# Supplementary material for: Identification and characterization of HAK/KUP/KT potassium transporter gene family in barley and their expression under abiotic stress
Source: BMC Genomics. 2021 May 1;22:317. doi: 10.1186/s12864-021-07633-y (PMC8088664; doi:10.1186/s12864-021-07633-y)
Supplement: Supplementary file 1 — Additional file 1. Protein sequences of 27 HvHAKs. [file 12864_2021_7633_MOESM1_ESM.docx]

**Protein sequences of 27 HvHAKs**

>HvHAK1

MASISDIETTNPGSLWELDQDLDVPMDEEASRLKNMYIEKKSSSVLLLRLAFQSLGVVFGDLGTSPLYVFYNIFPHGVDNDEDVIGALSLIIYTLTLIPLMKYVFVVLRANDNGQGGTFALYSLLCRHAKVSTIPNQHKTDEELTTYSRQTYEENSLAAKVKRWLEGHGYKKNCVLILVLIGTCTAIGDGILTPAISVLSATGGIRVQNPKMSTDVVVIVAVAILIGLFSMQHYGTDKVGWLFAPLVFLWFILIGSLGAFNIHKYNSSVLKAYNPVYIYRFLRRGKSEIWTSLGGVMLSITGTEALFADLCHFPVLAIQIAFTLVVFPCLLLAYTGQAAYIIVHKDHVVDAFYRSIPDAIYWPAFIIATLAAIVASQATISATFSIIKQALALGCFPRVSVVHTSKKFLGQIYIPDINWVLMILCIAVTAGFKNQIQIGNAYGTAVVIVMLVTTFLMVPIMLLVWKSHWILVVTFLVLSLMVEFPYFIACINKVDEGGWVPLAVAITFFIIMYVWHFCTVKRYEFEMHSKVSMAWILGLGPSLGLVRVPGIGFVYTELASGVPHIFSHFITNLPAIHSVVVFVCVKYLPVYTVPVEERFIMKRIGPKNFHMFRCVTRYGYKDIHKKHDDFEKMLLDRLLIFVRLESMMDGYSDSEDFTMSERKVQRSTNALLMSEKAGSDLSYSSHDSSIVLAKSPPTGNNSLTRYSSQTFGDELEFLNSCKDAGVVHILGNTIVRARRDSGIIKRIVVDHLYAFLRKVCREHSVIFNVPHESLLNVGQIYYI

>HvHAK2

MDCERGISPENIAGVYPDDDDPHGNPRHWRSYYKHVLLLAYQSCGVVYGDLSTSPLYVYKSTFAGPLRRFEDEETIFGVFSLVFWTVTLIPLLKYVFIVLSADDNGEGGTFALYSLLVRHAKFSLMPNQQAADEELSTYYKPGYAAHDTAILRALRRFLENHSKSRTCLLLTVLFGASLVIGDGVLTPAMSVLSSFSGLQVHSHTLSNGEVVILSCIVLVCLFTLQHWGTHRVAFLFAPVIVIWLLLLGGVGAYNIFAWNPRVFYALSPTYLVRFFMRTGREGWIALGGVLLSMTGTEAMFADLGHFTATSIRVAFVGLIYPCLVLQYMGQAAFLSKTPECDIHFIFFQSIPVRLFWPVMVIATLASIVGSQAVITATFSIVRQCMALGCFPRVKIVHTSSLIPGQIYSPEINWILMLVCLGVTVGFRDTMLIGNAYGMACAGVMVVTTLLMSLVIVFVWQLGFIMATLFLLAFGVVEVAYLSAALMKVPQGGWLPLAFSLLVVAIMYTWHYGTRLKHTFDVQNKVSLRWIHALGPSLGIVRVPGIGLIYSELATGVPAVFSHFVTNLPAFHQVLVFVCVKAVPVPHVCPEERHLVGRIGPRDFHMFRCVVRYGYKDLLGEDSDFENDLVLRIAEFVHMEAADNRPSDGAASAVEGRMAVVRRPSDLERTGRLLVQEPVDEESLVLRAATAATDGDKSDTLQSLQAMYEPESPAAAYGNRRQRVRFELSDLAGEHVDPEVKAELAAIVEAKHAGVAYIMGHSYIKARKSSNVFKKFAIDIAYNFLRKNSRGPAVALNIPHISLIEVGMIYYV

>HvHAK3

MSVEAADGAETQLARHDSLYGDAEKVAGDRRHGSGASWRQTVLLAFQSVGVVYGDIGTSRLYTISSTFPDGIRHPDDLLGVLSLVLYTLLLLPMLKYVFIVLYANDNGDGGTFALYSLISRYAKIRMIPNHQAEDAAVSNYMTAHEPSSQARRAQCVKKSLESSKAAKIALFTITILGTSMVMGDGTLTPAISVLSAVSGIREKAPNLTQSQVVWISVAILFLLFSVQRFGTDKVGYSFAPIISVWFVLIAGIGAYNLAAHDATVLRALNPRYMVDYFRRNGKEAWLSLGGVVLCTTGTEAMFADLGHFNIRAIQLSFSCIIFPSVALSYMGQASYLHKFPQDVGDTFYKSIPAAMFWPTFIVAIMAAIIASQAMLSGAFAILSKALSLGCFPRVEVVHTSNKYAGQVYIPEINFLIGAASIVVTLAFQTTANIGNAYGICVVMVFSITTHLMTIVMLLVWKTNVAFIAAFYVVFSLTELLYLSSILSKFAEGGYLPFCFSLVLMALMATWHYVHVRRYWYELDHVVPAAELAALLGRRDVRRVPGVGLLYSELVQGIPPVFPRLVDKMPSVHAVFVFMSIKNLPIPRVPAPERFIFRRVGPADHRMFRCVARYGYTDQIEAAKEFSAALLDGLKLFVHQEAAFFDDDNDDGALQLRGARAAATEEEKRFIDAELERGVVYLTGEADVVAAPGSSVLKRIVVNYVYSFLRKNLRESHKALAIPKDQLLKVGITYEI

>HvHAK4

MEPGKRETWRTTMLLAYQSLGVVYGDLSISPLYVYKSTFADDITHTDSNDEILGVLSFVFWTLTLVPLIKYVSIVLRAHDNGEGGTFALYSLICRHANVSLLPNRQLADEELSTYSLERPPEEVAHGSRVRRWLEGHRSLKTALLVMVMIGTCMVIGDGVLTPVISVFSAVSGLELSLSKHQHEYAVTPITCAIIVFLFALQHYGTHRVGFLFAPIILAWLICMSALGVYNIIYWNPQVYMALNPMYMLKFLRKTKKSGWMSLGGIVLCMTGSEAMFADLGHFSYSAIQLAFTSLVYPALILGYMGQAAYLTRHHNFDSSYQIGYYISVPEAVRWPVLVLAIMASVVGSQAIISGTFSIINQSQALSCFPRVKVVHTSAKVHGQIYIPEINWMLMVLCIAVTVGFRDTKHMGNASGLAVITVMLVTTCLTSLVMMLCWQRPPALALAFFVFFGSIEALYFSASLTKFLDGAWVPLLLALILVAVMFVWHHTTVKKYEFDLHNKVTMEWLLALCDRLGMVRVPGIGLVYTDLTSGVPANFSRFVTNLPAFHRVLVFVCVKSVPVPRVLPAERYLVGRVGPAGHRSYRCIVRYGYRDVHQDVDSFETELVESLASFIRLDALFRCSDARSDADYERENAFTVIGSNPLRRRISYDDTHDSASSVEIRVDSMTGSGTNTVELAAVPTAARVVKRVRFLVDPGSPEVEDKQMLEELHELCEAREAGTAFIMGHSHVKAKPGSSLLRRLAIGYGYNFLRRNCRGPDVVLRVPPASLLEVGMVYVL

>HvHAK5

MDEEIGAAARQGQWKYHKALSLLAFQSFGVVYGDLSTSPLYVFKSALSGLDDYSDEATVFGLFSLIFWTLTLIPLLKYVIIVLAADDNGEGGTFALYSLLCRHAKMSLLPNQQAADEELSTYYQPGVDRTAMSSPFKRFLEKHKKLRTCLLLFVLFGACMVIGDGVLTPTISVLAALSGLQDKDTGGLGNGWVVLIACVVLVGLFALQHRGTHRVAFVFAPIVVLWLLSIGIIGLYNIIRWNPRVCLALSPHYIVKFFKITGRDGWISLGGVLLAVTGTEAMFADLGHFTAASIRLAFVGVIYPCLVLQYMGQAAFLSKNMDAVHDSFYLSIPRTVFWPMFVLASLAAIVGSQSIISATFSIVKQCLSLGCFPRVKVVHTSRWIYGQIYIPEINWILMCLCLAVTIGFRDINIIGNAYGLACITVMFVTTWLMALVIIFVWKKNIMLALLFLIFFGSIEGAYLSASFIKVPQGGWTPIALAFVFMFIMYVWHYGTRRKYLFDLQNKVSMKWILTLGPSLGIVRVPGIGLIYTELVTGVPAIFSHFVTNLPAFHQILVFVCVKSVPVPYVPADERYLIGRIGPRQYRMYRCIVRYGYKDVQKDDENFENHLVMSIAKFIQMEAEEAASSRSYESSNEGRMAVIHTTDATGTGLVMRDSNEGTSLTRSSKSETLQSLQSIYEQESGSLSRRRVRFQIAEEEQVNPQVRDELSDLLEAKEAGVAYIIGHSYVKARKNSNFLKSFAIDYAYSFLRKNCRGPSVTLHIPHISLIEVGMIYYV

>HvHAK6

METGSSGGRRLPKTESAEMRWVVSGGASEDDEIDSSDDGDGGTDTPTAALGSRGGGGGYSDAEEDEEDALLRQRLVRTGPRADSFDVEALDVPGLYRHQEFTVGRSIVLALQTLGVVFGDVGTSPLYTFDIMFNKYPNTSKEDVLGALSLVIYTLILIPLLKYTLIVLWGNDDGEGGIFALYSLICRNAKASLLPNQLPSDTRISSFQLKVPSVELERSLRIKERLETSSMLKKLLLMLVLFGTSMVIADGVVTPAMSVMSAVNGLKVGISSVNEGEVVMISVAFLIVLFSLQRFGTSKVGLAVGPALFIWFCCLSGIGIYNIIKYGTEVLRAFNPIYIYYYFEKNPTQAWMSLGGCLLCATGSEAMFADLCYFSVRSVQLTFVCLVLPCLLLGYLGQAAFLMENLTENEQVFFLSIPSQVFWPVVFIATLAALIASRTMTTAIFSIIKQATALGCFPRLKIIHTSRKFMGQIYIPVMNWFLLVSCLAFVTTFGSINEIGNAYGIAELGVMMMTTILVTIIMLLIWQVNIIVVLCFLTLFLGLELFFFSSVLGSVADGSWVLLVFAAVLYLVMYIWNYGTKLKYETEVKQKLSMDLMMDLGCNLGTVRAPGIGLLYNELVRGVPAIFGHFLTTMPAIHSMIIFVCIKWVPVPVVPQNERFLFRRVCPKNYHMFRCIARYGYKDVRKENPQTFEQLLIESLEKFIRREAQERSLESDENGNTDSEEEVGSTSSRVLVGPNGSIYSLGVPLLAESAGVSNPNLGSSTSFDGSLDGTMDGRRSLDNELSFIHKAKECGVVYLLGHGDIRARKESFFAKKLVINYFYAFLRKNCRRGIATLSIPHTRLMQVAMQYMV

>HvHAK7

MAEQQQKQQQQGGDDEMLEAALEMPEVVQRQDSLYRDASRAGGASHHGHERWGKTLRLAFQCVGVLYGDIGTSPLYVYSSTFTAGVRHTDDLLGVLSLIIYSFILFTMVKYVYIALRANDDGDGGTFALYSLISRHAKVSLVPNQQAEDELHILDQEDPKSFSRRRGLATLQLASPAAHRAQRVKELLETSKPVRISLFLLTILATAMVISDACLTPAISVLSAVGGLKEKAPHLTTDQIVWITVAILVALFSVQRFGTDKVGYFFAPVVILWLLLIGGVGVYNLVKHDIGVLRAFNPKYIADYFRRNKKDAWISLGGILLCFTGTEALFADLGYFSIRSIQLSFGFGLVPSVLLAYAGQAAFLRKYPEEVANTFYRSTPTVLFWPTFVLAIAASIIGSQAMISCAFATISHSQALGCFPRVKILHTSKQYQGQLYIPEVNFLLGFAACVVTVAFKTTVVIGEAHGICVVLVMLITTLLLTVVMLLVWRMNAWCVALFFLVFMASESVYLSSVLYKFLHGGYIPVVISAVLMAVMIVWHYVHVMRYKYELERTVSPDKVREMLDGRDLRKVPGVGLFYTDLVQGIPPVFPHLIEKIPSIHAVLLFVSVKHLPVPHVDMSERFLFRQVEPREHKLYRCVARYGYRDPLEEAKDFATNLVERLQYYIRDVNLYGVDVDAKAGKVSYPSSRCDSMARSTRRSSMTMTMQQHYSSASYTESLALARARSTSSGATGRMNMNGMIMMPSASYTERERQGRSIYAEEMMTPAESFSELAMQVVPSGRYAASSQQLFQAAKMSLEEMAKIEEEQRYIEREMEKGVVYIMGENEVVARPHSSLLKKIIVNYVYAFLRKNCRQGDKMLAIPRSQLLKVGMSYEI

>HvHAK8

MSLQVEDPRSAETPAPLKRHDSLFGDAEKVSDSKHHGSQVSWMRTLSLAFQSVGIIYGDIGTSPLYVYSSTFPDGIRNRDDLLGVLSLILYTLIIIPMLKYVFIVLYANDNGDGGTFALYSLISRYAKIRLIPDQQAEDAAVSNYHIEAPNSQLKRAQWLKQKLESSKAAKIVLFTLTILGTSMVIGDGTLTPAISVLSAVSGIREKAPSLTQTQVVLISVAILFMLFSVQRFGTDKVGYTFAPVISVWFLLIAGIGMYNLVVHDIGVLRAFNPMYIVQYFIRNGKSGWVSLGGIILCVTGTEGMFADLGHFNIRAVQLSFNGILFPSVALCYIGQAAYLRKFPDNVANTFYRSIPAPMFWPTFIVAILAAIIASQAMLSGAFAILSKALSLGCMPRVRVIHTSHKYEGQVYIPEVNFLMGLASIVVTVAFRTTTSIGHAYGICVVTTFAITTHLMTVVMLLIWKKHVMFIMLFYVVFGSIELIYLSSIMSKFIEGGYLPICFALVVMSLMAAWHYVQVKRYWYELDHIVPISEMTMLLEKNEVRRIPGVGLLYTELVQGIPPVFPRLIQKIPSVHSIFIFMSIKHLPISRVVPTERFIFRQVGPREHRMFRCVARYGYSDTLEEPKEFAAFLVDRLKMFIQEESAFALVQDQEESGGAGDVSDALARPRRSTVHSEEAVQGQARVSSHSASGRMSFHTSLAVEEEKQLIDREVERGMVYLMGEANVTAEAKSSILKKIVVNHVYTFLRKNLTEGHKVLAIPKDQLLKVGITYEI

>HvHAK9

MSLEVENPPSIETTKRLERQDSLIGDAEKVSNIKGHGSEGNWTQVLHLAFQSIGIIYGDVGTSPLYCYSSTFPNGVKDKDDILGVLSLILYTLILIPMIKYVFIVLYADDNGDGGTFALYSLISRYSKIRLIPNQQAEDSMVSNYSIESPSLTLKRAQWLKEKLESSKAAKIALFTITILGTSMVMGDGTLTPAISVLSAVSGIKEKVPSLTETQIVWISVPILLMLFSVQRFGTDKVGYSFAPIISVWFVLIAGIGMYNIVVYEIAILRAFNPMHILYYFSRNGKEAWISLGGAILCVTGTEGMYADLGHFNITAIQISFNGVLFPSVALCYMGQAAYLRKFPEDVADTFYRSLPAPLFWPTFTVAILSAIIASQAMLSGAFAILSKALSLGCFPRVRVIHTSKHHQGQVYIPEVNFLMGLASVIITITFRTTTEIGNAYGICVVTVFSITTHLMTIVMLLVWKKNIIFILLFYVVFSSIEWIYLSSILSKFIQGGYLPFCFSLVLMALMVTWHYVHVMKYWYELDHIVPIDEVTALLEKHNVQRIPGVGLLYSELVQGIPPVFLRLVQKIPSVHSIFLFMSIKHLPIPHVAPVERFVFRQVGPREHRMFRCVARYGYTDEVEDSGHFARFLAERLKMFIEDENAFEVEKPGNEDANSPTGVLEGQTMPRKSARSVIHSEEVIEPPMSNHVGRISSYSLQTIEEEKQLIDAEMKRGVVYLMGSANVIAGPESPALKVVVVDYVYSFVRRNLAEGHKVLSIPKDQLLKVGITYEI

>HvHAK10

MASLSESEGTNRGGMWELDQNLDQPMDEEATRLKNMYREKKFSSLLLLRLAFQSLGVVFGDLGTSPLYVFYNAFPHGVDNDEDVIGALSLIIYTLTLIPLLKYVFVVLRANDNGQGGTLALYSLLCRHAKINTIPNQHKTDEDLTTYSRQTYEENSLAAKIKRWLETRAYKRNCLLILVLLGTCTAIGDGILTPAISVLSASGGIKVQNPNMSTDIVVLVAVIILIGVFSMQHYGTDKVGWLFAPMVLIWFILIGTVGALNIHKHGSSVLKAYNPVYIYRYFRRRGNSSNTWTVLGGIMLSITGTEALFADLCHFPVLAIQIAFTCIVFPCLLLAYTGQAAYIIANKKHVNDAFYRSIPDAIYWPAFVIATAAAIIASQATISATYSIIKQALALGCFPRVKVVHTSKKFLGQIYIPDINWLLLVLCIAVTAGFKNQSQIGSAYGTAVVIVMLVTTFLMVPIMLLVWKSHWVLVVTFIVLSLMVELPYFWACILKIDQGGWVPLVIAIAFFVIMYVWHYCTVKRYEFEMHSKVSMAWILGLGPSLGLVRVPGIGFVYTELASGVPHIFSHFITNLPAIHSVVVFVCVKYLPVYTVPVEERFLVRRIGPKNFHIFRCIARYGYKDLHKKDDDFEKMLFDCLTLFIRLESMMDGYSDSDEFSLPEQRTEGSINTAFLADKTANTMCSNGDLSYSSQDSIVPVQSPLGVNNLLTYSSQTNRTVSNEVEFLNRCRDAGVVHILGNTIVRARRDSGIIKKIAVDYFYAFMRRICRENSVMFNIPHESLLNVGQIYYI

>HvHAK11

MAAESSAAAGMRKAPSMEWRWVSAGEEEDDELEGRRGEGGPAAVGAAGRGGSFESEDEEDNVDYEDEDEEQREARQRLIRTVPSVDWFDVEGNEVSGAQQLEDPEEFDFGRTVFLALQTLAVVFGDIGISPLYTFDVMFNKYPILEEEDVLGALSLVLYTLILMPLVKYVLVVLWANDDGEGGIFAMYSLICRNAKVSLIPNQVQAQAEKRMSSFRLKLPTDELERSIKVKEKLESSLLMKKLLLGLVLFGTAMFISNGVITPAMSVLSAVSGLKVGIPKASQDVVVMISIALLIVLYSLQRYATSKIGFVVGPCLLIWFCCLGGIGICNLSRYGPAAFKAFNPLYIIYYFGRNPFQAWLSLGGCLLCVTGSEAIFSNLCHFPVRFVQSMFVLLVLPCLVLAYLGQAAFLIANQKTPEHIFFASIPRNAFWPVFLLANLAALIASRTMTIAIFQCLKQSISLGCFPRLKIVHTSRKFMAKIYIPVVNWFLLASCLGFILLFRSTSDVGNAYAIAEIGVMIMATIYVTIIMLLIWETNIIKVMSFLITFLSLELIFFSSALSSVGDGGWALLVFASGLLMIMFIWNYGTKLKYDSELKQKLSKDLMRKLGPNLGTMRAPGLGLVYSEIVTGVPAIFGHFLTALPAIHSIIVFVCVRNVPVPAVPQSERFLFQRVCSRGYHMFRCIARYGYKDKKQEHHNTFERLLIEGLEKFIQREAVELSLQSEDDVDSDEEPSTPGQIITAPNGSVYSLDAPLLVDFTPSVDSIPETPSCSTPQDPALDYTQNLELELAFIKQAKQSGAVYLIDNPIVKARKDSWFFKKLTINYFFAFLRNNCRRAIVSMSIPHSNLLQVRLTSYV

>HvHAK12

MAEPLKTNGNGAAEGGSAFASVKVPPPPSPPRRLQRFDSLHMEAGMIPGGHSYAAKVGWPTTLHLAFQSLGVVYGDMGTSPLYVFSSTFTGGIKDTDDLLGVMSLIIYTVLLLPLMKYCFIVLRANDNGDGGTFALYSLISRYARISLIPNQQAEDATVSHYKLESPTNRVKRAHWIKEKMENSPKFKVILFLVTILATSMVIGDGVLTPCISVLSAVTGIKQSAKSLTQGQIAGIAIGILIVLFLVQRFGTDKVGYTFGPVIFIWFILIAGIGIYNLITHDTGILKAFNPKYIVEYFQRNGKDGWISLGGVILCITGTEAMFADLGHFNVRAIQIGFSAVLLPSVLLAYMGQAAYLRIYPEDVADTFYKSLPGPLYWPTFVVAVAAAIIASQAMISGAFAIIAQSQVLGCFPRVRVTHTSKKYHGQVYIPEINYALMILCVAVTAIFQTTDKIGNAYGIAVVFVMFITTLLVTLVMAMIWKTSLLWIALFPIIFGGAELLYLSSAFYKFVEGGYLPLGFAAILMLIMGTWHYVHVHRYKYELKNKVSNNYVAELATRRNLARLPGIGVLYSELVQGIPPILPHLVEKVPSIHSVLVITSIKFLPISNIETNERFLFRYVEPREYRVFRCVVRYGYNNKVEDPREFENLLIGNLKQFIHQESLYSESSHSLAGEDNAMEESGDAMEPSVEVQDARLPKRFVDGITASPVNGCMDEIEFIQRGMDDGVVHLLGETNVVAEQNAGLVKKIIVDYAYNFMRKNFRQPEKITCVPHNRLLRVGMTYEI

>HvHAK13

MDMERRAGENGDVVLEINSPGADGHQQGGDGGANGGGDAARRTLSFSQAYKMRHRTPQAFTVSQTLLLSFQSLGIVYGDLGTSPLYVFPSVVLPGAGERDFLGILSLILWTLTLMSLVKYVLIVLRADDHGEGGTFALYSLLRQHVSFKTGSTPAQVTRLPSDLQLRFHGKKRRPEPSRVQRFLEGSAAAQSVLTYVVLVGTSMVMGDGALTPAISVLSAVQGIQSRSPKIEQKHVVMLSVVILLLLFLFQRMGTSRVSFSFSPIMLVWFASIAMIGLYNIVVYYPPVLKALNPYYIYCYFARNGAVGWEQLGAVILCITGAEAMFADLGHFNKRSIQVAFSTVVYPSLILAYSGQAAYLIKNPDHLSTAFYSSIPGPLFWPMFVVSTLAAIVASQSLISASFSIIRQSIVLDYFPRATVKHTSDKYEGQVYCPEVNYLLMVFCVLITIGFQGGPEIGHAFGVAVIWVMLITTALMTVVMVVIWDVHPVIAAAFFAVYVAVEGLYMSSLMNKMAQGGWVPFAITAFFLVITVSWTYGRKKKGEYEAGHMISGNELAAVVARSARVPGVCFFFTDLMNGIPPIVRHYAEHTGCLRELLLFVTVRRLPVTSVLPEERFLVASEEEVPPGVYRSVVQYGYMDKQDMEGEEFLESVLAALKEVARTAEEAAMMDRACRSGVSVVIGRTILTASGGKHVHGWFRRFVVNHMYRFLQKNFNSGVSNLKLDHEKTMQVGMRYSVKLD

>HvHAK14

MDAEAGAVAAAQELPWRQHYRNLLLLAYQSFGVVYGDLSTSPLYVYKSTFSGRLGQYQDEQTVYGVLSLIFWTFTLVPLLKYVIIVLSADDNGEGGPFALYSLLCRHAKLSLLPNQQAADEELSTYYRDGFAAQHGSSPWLRRFLEKHKTVKTGLLVVVLCAASMVIGDGVLTPAISVLSSMSGLQVRATGLQERSVVLLSCIVLVGLFSLQHRGTHKVAFMFAPIVIIWLLCIGGIGLYNIVHWNPKIYQAISPYYIVKFFRTTGTDGWIALGGILLSMTGSEAMFADLGHFTSASVRLAFITIIYPCLILQYMGQAAFLSKNMLHMRTSFYDSIPGPVFWPVFVVATLAAVVGSQAVISATFSIVKQCHALGCFPRVKIVHTSRWIYGQIYIPEINWILMVLCVAVTVAFGDTTLIGNAYGIACMTVMLITTFFMAFIIIFVWQKNIIFALLFLLFFGSIETVYLSSSLMKVHQGGWVPLVLAFIFMSVMFIWHYGTKRKYQFDLQNKVSMRSILSLGPNLGIVRVPGIGLIYTELVTGVPAIFTHFVTNLPAFHEVLVFLCVKSVPVPYVQPDERYLVGRIGPRAYRMYRCIVRYGYKDVQRDDENFENMLVMSIARFIMMEAEDVSSSASYDIANEGRMAVIRTTDDAGTPLGMRDLGGLAESISTTRSSKSESLRSLQSSYEQESPSANRRRRVRFELPNEDAMDQQVKDELLALVEAKHAGVAYIMGHSYIKARRSSNFLKKFAVDVGYSFLRKNCRGPSVSLHIPHISLIEVGMIYYV

>HvHAK15

MSVEADGAAGAERVLRRDSLYGDAEKVTNDKHHGSGASWRQTLQLAFQSIGVVYGDVGTSPLYVYSSTFPDGIRHPDDLLGVLSLIIYTLILLPMLKYVFIVLYANDNGDGGTFALYSLISRYAKIGMIPNQQAEDASVSNYSIEEPNSKMRRAQWVKQRLESSKAAKIALFTITILGTAMVMGDGTLTPAISVLSAVGGVREKAPNLTQSEVVWISVAILFLLFSVQRFGTDKVGYSFAPIISVWFILIAGIGAYNLAAHDVTVLRALNPKYIVDYFGRNGKEAWVSLGGVVLCITGTEAMFADLGHFNIRAIQLSFTFILFPSVALCYMGQASYLRKFPQNVGDTFYKSIPAAMFWPTFIVAIMAAIIASQAMLSGAFAILSKALSLGCFPRVKVVHTSKKHSGQVYIPEVNFLIGAASIVVTLAFQTTTNIGNAYGICVVTVFSITTHLMTVVMLLIWKKNFAFVVVFYVIFGLAEFLYLSSILSKFVEGGYLPFCLSVVLMALMATWHYVYVKRYWYELDRVVPADQLTALLARRNVRRVPGVGLLYSELVQGIPPVFPRLVDKIPSVHAVFVFMSIKNLPIPRVALPERFIFRRVGPAEHRMFRCVARYGYTDQIEGTKEFSTFLIEGLKMFVHDEAAFSCQHTDDDGDNNNNDNDARRVAQAAIAEEEKRFIDTEVERGVVYLMGEADVAAAPGSSALKRIVVNYVYTFLRKNLSESHKALSIPKDQLLKVGITYEI

>HvHAK16

MDLEFGRGMRSPQRDSWRTTMLLAYQSLGVVYGDLSISPLYVFKSTFAEDIQHSDTNEEIFGVLSFVFWTLTLIPLIKYVSIVLRADDNGEGGTFALYSLICRHANVSLLPNRQIADEELSTYKLERNPETVDKTRVKVWLEKHKNLHTALLVMVLIGTCMVIGDGVLTPAISVFSAVSGLEFSLSKDHHEYAVIPITCVILAFLFALQHFGTHRVGFIFAPIVLAWLFCMSALGLYNIIHWNPHVYQALNPYYMFKFLKKTRKYGWMSLGGILLCMTGSEAMFADLGHFSYSAIQLAFTSLVYPALILAYMGQAAYLSKHHDFYSNSQVGFYIAVPDKVRWPVLVLAILASVVGSQAIISGTFSIINQSQSLSCFPRVKVVHTSEKIHGQIYIPEINWLLMILCIAVTVGFRDTKHMGNASGLAVITVMLVTTFLTSLVIMVCWHKPPLLALGFLLFFGSVEALYFSASLIKFLEGAWLPILLALILMAVMLVWHITTIKKYEFDLQNKVTLEWLLALGDKLGMVRVPGIGLVYTDLTSGVPANFSRFVTNLPAFHKVLVFVCVKSVPVPYVFPAERYLVGRVGPPGHRSYRCIVRYGYRDVHQDVDSFETELIESLAMFIKLNASYRCSEVSEGEQLEEWEPGLTVIGSNTLRDHASYDLQDSVQHSAASVEMRPAATSPGGTGLELAAESNSPKQVRFFIDNLVVSPEADKQVAEELEALAAAREAGTAFILGHSHVQSKPGSSVLKKLTVVGYNFLRRNCRGPDVALRVPPASLLEVGMVYVL

>HvHAK17

MSTQAEEPQSTETVPAPLKRHDSLWGDAEKVSHSNHHGSRVSWVRTLSLAFQSVGIIYGDIGTSPLYVYSSTFPDGIKNNDDLLGVLSLIIYTLIVIPMLKYVFIVLYANDNGDGGTFALYSLISRYAKIRMIPDQQAEDAAVSNYRIEAPNSQLRRAQWAKQKLESSKAAKIALFTLTILGTSMVIGDGTLTPAISVLSAVGGIREKAPSLTQTQVVLISVAILFMLFSVQRFGTDKVGYTFAPVISVWFLLIAGIGMYNLVVHDIGVLRAFNPIYIVQYFQRNGKEAWVSLGGVILCVTGTEGMFADLGHFNIRAVQISFNFILFPGVGLCYIGQAAYLRKFPENVANTFYRSIPAPMFWPTFIVAILAAIIASQAMLSGAFAILSKALSLGCMPRVQVIHTSHKYEGQVYIPEVNFIMGLASIIVTVVFKTTTSIGHAYGICVVTTFIITTHLMTVVMLLIWKKHVIFIALFYIVFGSIEVIYLSSILSKFIEGGYLPICFALVVMSLMAAWHYVQVKRYWYELDHIVPTSELSVLLKKNDVRRIPGVGLLYTELVQGIPPVFPRLIERIPSVHSIFMFMSIKHLPIPRVLPAERFLFRQVGPREQRMFRCVARYGYSDTLEEPKEFVIFLMNGLKMFIQEESAFAHNEVAEIATGGEVSGRSTSNVVHSEEVVQARVSSHSSGRIGSFHSNRTVEEEKELIDKEVEHGMVYLMGEANVSAKANSSVFKKMVVNYVYTFLRKNLTEGHKALAIPKDQLLKVGVTYEI

>HvHAK18

MDDGGIQEEEEQPSASARLLRPKRSGGSSRWVDASEVDSSESAHLSLEDERSPPWTLSAADEAEVLTATGGPELSRRSSSGFRRRLGKRPKRVDSLDVEAMTVRGAHGHSIQDVSLMSTVAMAFQTLGVVYGDMGTSPLYVFSDVFSKVPIKSEVEILGALSLVMYTIALIPFAKYVFIVLKANDNGEGGTFALYSLICRYAKVSLLPNQQRVDEDISSFRLKLPTPELERALFVKDCLEKKPLFKNILLFLVLMGTSMVIGDGILTPSMSVMSAVSGLQGQVAGFDTDAVVIVSILVLLLLFSVQRFGTGKVGIMFAPVLALWFLNLGSIGIYNIIKYDTSVVRALNPMYIYYFFKMNGIKAWSALGGCVLCITGAEAMFADLGHFTVKSIQLAFTAVVFPCLLIAYMGQAAYLMKHPLDVERIFYDSVPEVLFWPVFVIATLAAMIASQAMISATFSCIKQAMALGCFPRIKIIHTSKKVMGQIYIPVMNWFLMVMCIIIVATFRSTNDIANAYGIAEVGVMMVSTALVTLVMLLIWQTNLVLVLCFPIFFGAMEFIYLTAVMSKLLEGGWLPLAFSSLFLCIMYTWNYGSVLKYQSEMRGKISLDFILDLGSTLGTVRVPGIGLVYNELVQGIPSIFGHLLITLPAMHSTIVFVCIKFVPVPYVPLEERFLFRRVGQKDYHMFRCVARYGYKDVRKEDHGSFEHLLVESLEKFLRREAQELALEVSAMEAERDDVSDVSEIVQSPAAPAEDLHTPLLSDQRPGDDNEMLGMEGSVPLLPSSSMSAEEDPSLEYELAALREAMASGFTYLLAHGDVRARKQSFFTKKFIINYFYAFLRRNCRAGTATLKMPHSNIMRVGMTYMV

>HvHAK19

MSTGAHMDLEADPGVLPAATPPPPAAHKETNVGNVRKDLFLAYKTLGVVFGGLVTSPLYVFSTMHMSSPTEADFLGIYSIMFWTLTLIGVVKYVGIALNADDHGEGGTFAMYSLLCRHANMGILPSKRVYSAEEQLLHNQSKTAKRPSNLGKFFERSLTARRVLLFMSILGMCMLIGDGVLTPAISVLSAIQGLRAPFPAVTQPIVEFLSAAILIGLFLVQKFGTSKVSFLFSPIMAAWTFTTPIVGIYSIFRYYPGIFKAISPHYIVHFFLRNKKEGWKMLGATVLSITGAEAMFADLGHFSKKAIQIAFLSSVYPSLILTYAGQTACLINHVKDTDQENTGKVFDDAFYKFIPRPVYWPMFVIATLAAIVASQSLISATFSVIKQSVVLDYFPRVKVVHTSDENEGEVYSPETNYILMVLCVGVILGFGGGQAIGNAFGLVVIMVMLITSIMLTLVMIIIWRTPPVLIALYFVPFVVMEGSYVSAVFTKFTEGGWLPFAISMILALIMFVWYYGRQKKTEYERANKITAERLGELLAMPEVQRVQGLCFFYSNMQDGLTPILGHYISNMSSLHSVTIFVTLRYLLVPKVDARQRITVRRLGPRGVYQCTVQYGYADNLSLKGGDDLVGHVMNCLKQHIEASADGQSSPFSTEEEAADLEAARSAGVVHVRGKMRLYVGDDAGCFDKVMLRFYEFLHSICRSALPALGVPLQQRVEIGMLYKV

>HvHAK20

METVSTNEDTGKGAMWELEKSLDQPMDAEAGRLRNMYREKTYPTVLMLQLAFQSLGVVFGDLGTSPLYVFYNIFPNEIEDTEQIIGALSLIIYSLTLIPLVKYVFIVLRASDNGQGGTFALYSLLCRHAKISIIPNQHKTDEDLTTYSRQTYDEKSLAAKIKRWLEGHQFRKNVILILVLFGTCMAVGDGILTPAISVLSATGGIKVEEPRMRNDVVVIVSVMILIGLFSMQHYGTDKVSWLFAPIVFVWFILIGVLGAVNIYTYDRSVLKAFNPIYVYRYFKRGKTSWASLGGIMLSITGTEALFADLSYFPVQAIQIAFTTVVFPCLLLQYTGQAAYIATHKDKVSHSFYFSLPERILWPAFVVATAAAIVSSQATISATYSIIKQALAVGCFPRVKIIHTSKKYLGQIYSPDINWILLILCIAVTAGFKNQSQIANAYGTAVIMVMLVTTFLMIPIMLLVWRSHWALVLLFTVLSLVVEIPYLTAVMKKIDQGGWVPLVFAAAILLIMYVWHYGTLKRYEFEMHSKVSMAWILGLGPSLGLVRVPGIGLVYTELASGVPHIFSHFITNLPAIHSTLVFVCVKYLPVYTVPLDERFLVKRIGPKNFHMFRCVARYGYKDIHRKDDDFEKMLFSSLLLFVRLESMMEEYSDSDDYSALDQQELIDEASNDARSAADLSYASRDSIVPVRSPNLPGSMSSAQTTTATLGFETVGDEVAFLNSCRDAGVVHILGNTVIRARRDSGPLKKLAIDYLYAFLRKICRENSAIFNVPHESLLNVGQVFYV

>HvHAK21

MSAEAADGADTQLARHDSLYGDAEKVAGDRRHGSGASWRHTVLLAFQSVGVVYGDIGTSPLYTISSTFPDGIRHPDDLLGVLSLVLYTLLLLPMVKYVFIVLYANDNGDGGTFALYSLISRYAKIRMIPNHQAEDAAVSNYTTAHEPSSQARRAQWVKKMLESSNAAKIALFTITILGTSMVMGDGTLTPAISVLSAVSGIREKAPHLTQSQVVWISVAILFLLFSVQRFGTDKVGYSFAPIISVWFVLIAGIGAYNLAAHDATVLRALNPRYMVDYFRRNGKQAWLSLGGVVLCTTGTEAMFADLGHFNIRAIQLSFSCIIFPSVALCYMGQASYLHKFPQDVGDTFYKSIPAAMFWPTFIVAIMAAIIASQAMLSGAFAILSKALSLGCFPRVEVVHTSNKYAGQVYIPEINFLIGVASIVVTLAFQTTANIGNAYGICVVMVFSITTHLMTVVMLLVWKTNVAFIAAFYVVFSLTELLYLSSILSKFAEGGYLPFCFSLVLMALMATWHYVHVRRYWYELDHVVPAAELAALLGRRDVRRVPGVGLLYSEIVQGIPPVFPRLVDKMPSVHAVFVFMSIKNLPIPRVPAPERFIFRRVGPADHRMFRCVARYGYTDQIEAAKEFSAALLDGLKLFVHEEAAFFDDDNDDGALQLRGARAAAAEEEKRFVDAELERGVVYLTGEADVVAAPGSSVLKRIVVNYVYTFLRKNLRESHKALAIPKDQLLKVGITYEI

>HvHAK22

MDLELAHGAGAPRKRGESWGAVLLLAYQSLGVVYGDVATSPLYVFKSAFAGDDITHSEGNEEIYGVLSFVFWTLTLISLLKYVLIVLRANDGGEGGTFALYSLICRHVRAGLLPGGGTSDDLMAEDKDAAARRGAVSRARTVLERYRVLQRLLLLFALLGTCMVIGDGVLTPAVSVFSAVSGLELSMERAQHKYVGLPVTCAILICLFALQHYGTHRVGFLFAPIVCIWLLCISTIGLYNIIYWNHHVYRALSPYYMYQFLKKTQKGGWMSLGGILLCVTGSEAMYADLGHFSQRSIQIAFVSVVYPALVLAYMGQAAYISQHHSFEKNSYHIGFYVSVPEKLRWPVLVIAILASVVGSQAIITGTFSIIKQCSALSCFPGVKIVHTSSTVHGQIYIPEINWILMILCLAVTISFNNTKHLANAQGLAVITVMLVTTCLMSLVIVLVWNKSIFIALGFLIFFGSIEVLYFSASLVKFHEGAWVPITLSFIFMVVMSVWHYGTIKKYEFDVQNKVSVNWLLNLGPSLGIVRVRGIGLIHTELMSGIPAIFSHFVTNLPAFHQVLVFLCVKSVPVPHVEPEERFLVGRIGPKEYRLYRVIVRYGYRDVQQDDLEFEKELINSIAEFIRSGGADQNGFVEGSEKLSSISSGAIPLWEEDGDGEADGSASPNKEINQQTVAPQRRKARFVLPKSAQVDAEVRSELQDLMDAREAGMSFILGHSHMKAKSGSSFVKRIVINFFYEFLRRNSRGPSYAANIPHASTLEVGMVYQV

>HvHAK23

MAMGSARSSLEIVPHSGGDVEVPPADVPRQDSLYRDATRPAHGGHHGQDNWVRTLRLGFQCVGILYADLGTSPLYVFSNTFKYGVGHEDDVLGVLSLIIYSFLLFAMVKIIFIALYANDDGDGGTFALYSLISRYARVALIPNQQAEDDLVSTHRYLSATGRRAQWMKNLLETSKPAKLTLFFLTIFATALAISDCMLTPPISVLSAVNGLKLRAPHLTTDQIVWITVGILILFFAVQHLGTDKIGYTFAPLVVVWLLLIAGIGLYNLIKYDIGTLRAFNPKYIFDYFRRNKKKGWVSLGEILLCFTGTEALFADLGYFSIKSIQLSFSFGLLPSVLLTYIGQAAYLRKHLDMQISNAFFNSIPSTLFWPTFVLALLASVIGSQAMVSCAFATMSHLQTLSCFPRVKILHTSRRYSGQLYIPEVNFFLCVASCIVTLSFRTTGFIAKAHEICVALVMVITTLLMTIVMLLVWKVNIWWIAAFFAVFMSTETVYLSAVLYKFTQGPYFPLAMSAVLMVIMIVWHYVHVKRYKYELQHTVSPDEVRHLLERHDLKRVPGLGLFYTELVQGIPPIFPHLIEKIPTVHSVIVFISVKHLPIPHVDVQERFLFRQVEPKESMVFRCVARYGYRDTLEMAGDFVATLVEYLQYYVRDLSLYCTAEPLRTSYPSIRIDSFRWEKKPSGHGHGIHAEEMLTPIQSFSELTMHQVGMSNRLPQFQTAKMNLEEMLRIEEDQKLIQREVDNGVVYILGETEVVAKPHSNLLKKIAVNYIFDFLRKNSRKGEKMLSIPRGQLLKVGITYEI

>HvHAK24

MDAESGGGAARRKVSRPWSAELLLAYQSLGVVYGDVATSPLYVFKSAFAGGDIEHSAGNEEIYGVLSLVFWTLTLIPLLKYVLVVLRADDHGEGGTFALYSLICRRVRAGLLPDGEDLAGRREGGAAPPAPLSAVRAALERHRVLQRMLLLLALLGTCMVIGDGVLTPAVSVFSAVSGLELELDNEQHEYILLPVTCAILVGLFTLQHYGTHRVGFLFAPIVCLWLLCISIIGLYNIIHWNPHVYRALSPYYMYKFLQKTQTGGWMSLGGILLCVTGSEAMYADLGHFSQSSIKIAFTSLVYPALILAYMGQAAYISRHHNFENINHIGFYVSVPEKIRWPVLVIAILAAVVGSQAVITGTFSIIKQCCSLSCFPRVKIVHTSSTVHGQIYIPEINWILMILCLAVTIGFRDTKHLTNAQGLAVITVMLVTTCLMSLVIVLCWNKSILFSLAFLLFFGAIEVLYFSASLVKFREGAWVPVMLSLFFMIMMCVWHYGTIKKYEFDVENKVSISWLLNLGPSLGIVRVRGIGLIHTELMSGIPAIFSHFVTNLPAFHQVLVFLCIKSVPIPHIRPEERFWVGRVGPKQYRLYRVVVRYGYRDVPKDDIEFEKDLVCSIAEFIRCGDSDDQNGFLDGATDHTCERLSSISKGLPFQEEDGSEINGSDSSILSTDKEMYQNTIGPKAKRVRFVLPKDAQIDSEVRSELQELTDAREAGMSFITGRAHMKAKSGSGLVKKIAINYIYEFLRRNSRGSVSAANIPHASTLEVGMVCQM

>HvHAK25

MEHQPPADHVVVQLSAAAVAAVDERSSTNHDDSAGATRRTFSQYYKMEHRKAPDFKWWQIGVLSYQSLGIIYGDLGTSPLYVFSTVTLPDPGEEDFLGILSLILWTLTLIGLLKYTFVVLHADDHGEGGTFALYSLLRQHVNFSGSMPMPVTRLASDINLKFHSKKKKLPSKMREFLERSTTAQSVITYVVLIATSMVMGDGALTPAISVLSAVQGIQSRSPKITQDHVVILSVIILIILFLFENYGTSKVSFAFSPIMLLWFLFVSLIGLYNIIEYYPSVLKAASPLHIITFFARNKRKAWEQLGAIVLCITGAEAMFADLGHFNKSSIQLGFSAVVYPSMILAYSGQAAFLIKNPSKLSTTFYSSTPELLFWPMFIIATLSAIVASQALISASFSIIRQSIALGCFPRVTMKHTSEKYEGQVYSPEVNYFLMITCILITYGFKGGPQIGQAFGTVVIWVMLFTTTLMTVVMVVIWQTNIIAVGLFFVVFFSIEGIYMTSLLNKVLQGGWVPFANTAFFLAITISWTYGRRKKNEYDAANLLGKQEFIKIVTGSSQVPGICIFCTDLMNGIPPIVRHYVKHTGSIREVMVFVTVRILPVRSVLPEERFLVDKLDHVGVYRCILQYGYMDNHNMDDDDFVVLVVASLKQIADNDEILLLDSAFTNGTSFVIGRTILKMSITRNCFKRFVINSLYRFLQKNFRSNMSSLKIAPGKTLQVGMHYEI

>HvHAK26

MKSSPSMDPEAAAARGTPPENTEGRRGEGKKRLPWRMTLSLAYQSLGVVYGDLSTSPLYVYKAAFADDIQHSETNEEILGVLSFVFWTLTLVPLLKYVCVVLRADDNGEGGTFALYSLLCRHARAALLPPGRGAEPGDEDQFSDAAGATAKKYLEYDNADALGGRGGGAAASVRRVLERHKVLQRVLLVLALVGTCMVIGDGVLTPAISVFSAVSGLELSMEKGHHKYVELPLACFILVCLFALQHYGTHRVGFIFAPIVIAWLLCISMIGVYNIVKWEPQVYQALSPYYMYKFLKKTQRGGWMSLGGILLCVTGSEAMFADLGHFNQLSIQIAFTCMVYPSLILAYMGQAAYLSKHHILEGDYRVGFYVSVPEIIRWPVLAIAILAAVVGSQAVITGTFSMIKQCTSLGCFPRVKIVHTSAQVHGQIYIPEINWILMILCLAVTIGFRDTKHLGNASGLAVITVMLVTTCLMSLVIVLCWHKSIFLAIGFIVFFGTIEALYFSAALIKFKEGAWVPIVLAFVFMMVMCIWHYGTIKKYEFDVQNKVSINWLLGLSPNLGIIRVRGIGLIHTELDSGIPAIFSHFVTNLPAFHQVLIFMCIKNVPIPHVSPNERFLVGRIGPKEYRIYRCIVRYGYHDVQMDDQEFEKDLVCSVAEFIRSGGGASKANGLTPDVADRDEERMTVVASGRMRMLEEEGLGGASASGSTVGPSRAPRGEREIRSPSPTPTPTPTPTPAMGVRKRVRFVLPASTPRPNAGVEEELRELTDAREAGMAFILGHCYVKAKTGSSFLRRLVINFGYDFLRRNSRGPSYAVTVPHASTLEVGMIYYV

>HvHAK27

MDVEGGAVQEPRKKASWGWQKGTLLLAYQSFGVVYGDLCISPVYVYKNTFSGKLRLHEEDEEILGVLSLVFWSLTLIPLLKYIILVLGADDNGEGGTFALYSLMCRRSRMGLLNSLHAGHGSVSSFNNDELCKETRSSLAIRGFFEKHHSLRVVLLLFVLMGTSMVIGDGVLTPTMSVLSAVSGLRIKFPELHENYTVLIACVVLVGLFALQHYGTRRVGFLFAPILLSWLACIGGIGIYNIFRWNPTVVRALSPYYIYNFFRKAGRDGWSSLGGIVLCITGAEAMFADLGHFSKLSLRLGFTIVVYPCLVLAYMGEAAYLSKHREDLQSSFYKALPDRVFWPVLIIATLATAVGSQAIISATFSIISQCRALGCFPRIKVVHTSSHVHGQIYIPEVNWTLMSLCLAVTIGFRDTEMIGNAYGLAVILVMFATTCLMFLVITTVWNRSVLWAALFAAGFGSVELMYLSACLAKVPHGGWLPLLLSLATLLVMSAWHYGTAKKQEYELQNKVCLDHFLGLSSGMGLVRVPGVGFVYSDSVAGVPPMFAHFVTNFPAFHRVLVFVSLQTLTVPKVPPEERFLVGRIGRPEHRMFRCVVRYGYKEGRWDHFNFENQLLVKVLEFLQLQQADGDGERCSTGSGEMSVIPAAPSQAVVDALASMSSGEIDYYAGSGAKKVRFEELPAAWRREETMSEVRELLEEREAGVSYMIGHTCVFAHESSSAVKKFAVNVVYGFLRRNSRRPAVVLGIPHTSLIEVGMVYRV
