# Supplementary figures and images for: Identification and characterization of HAK/KUP/KT potassium transporter gene family in barley and their expression under abiotic stress
Source: BMC Genomics. 2021 May 1;22:317. doi: 10.1186/s12864-021-07633-y (PMC8088664; doi:10.1186/s12864-021-07633-y)

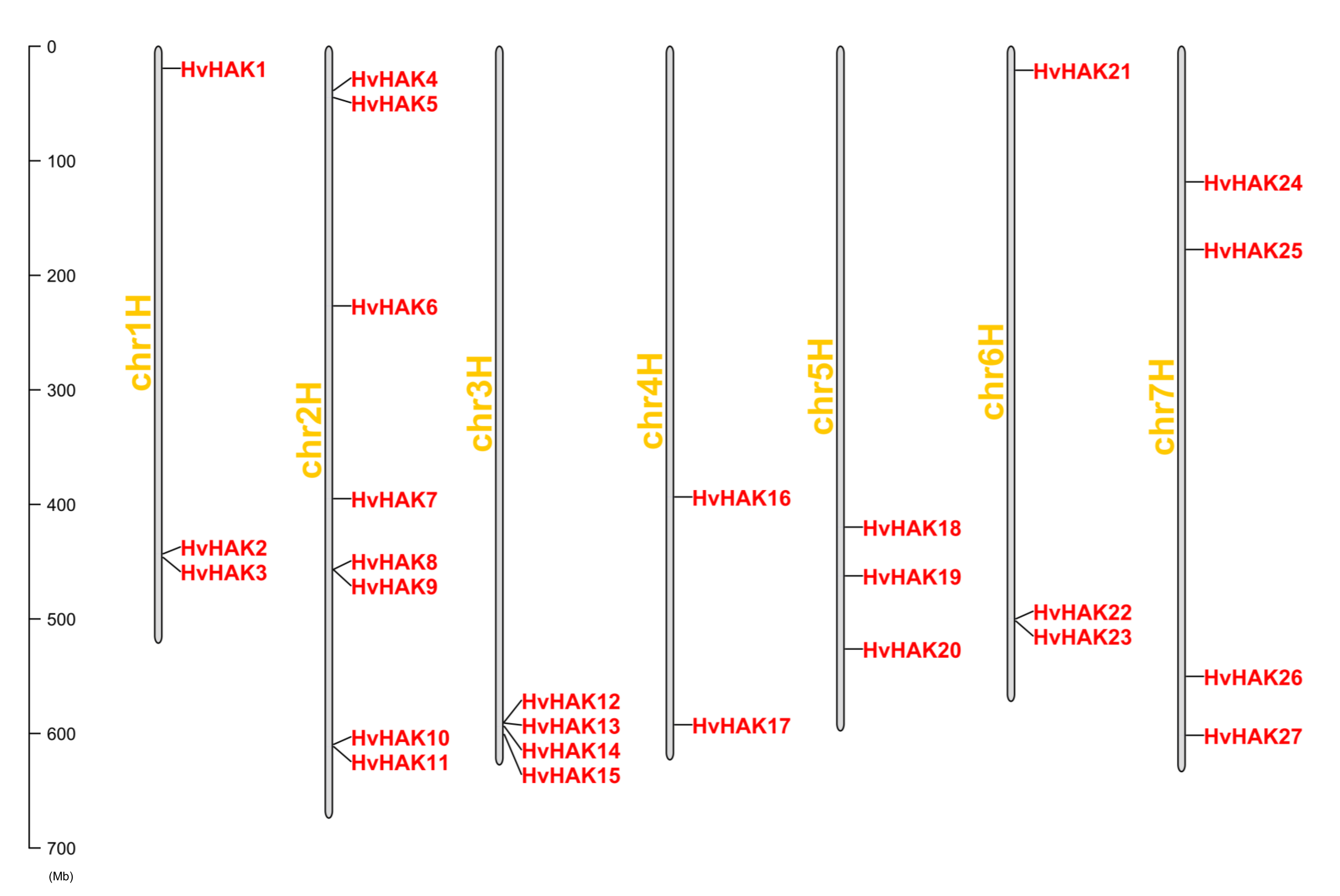

Supplement: Supplementary file 2 — Additional file 2 Chromosomal distribution of HvHAK genes. Chromosome names are displayed on the left side of chromosomes. HvHAK gene names are indicated on the right side of chromosomes. [file 12864_2021_7633_MOESM2_ESM.tif]

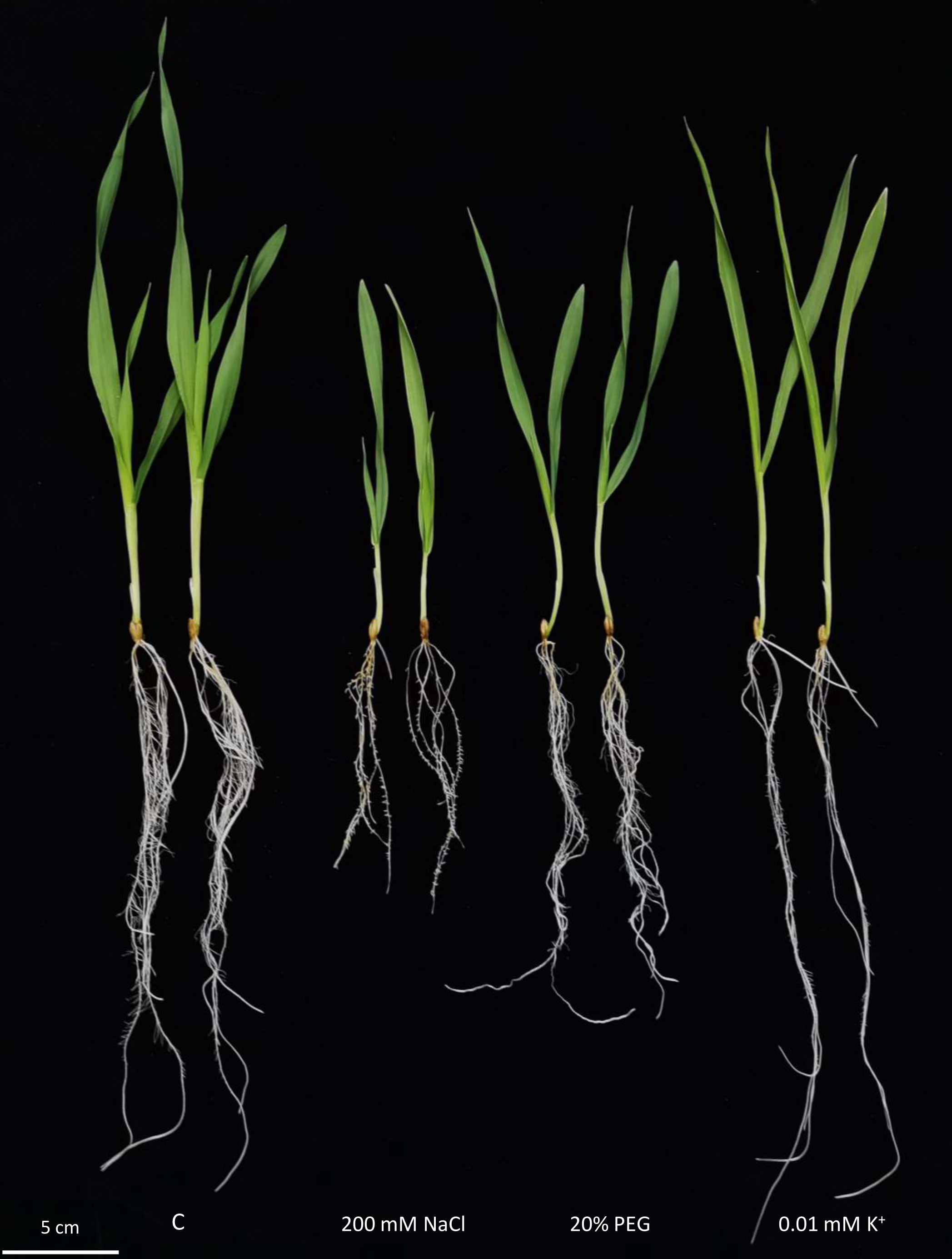

Supplement: Supplementary file 6 — Additional file 6. Phenotypes of barley seedlings after abiotic stress treatments for 6 d. [file 12864_2021_7633_MOESM6_ESM.tif]
